# An ancestral 10-bp repeat expansion in *VWA1* causes recessive hereditary motor neuropathy

**DOI:** 10.1093/brain/awaa420

**Published:** 2021-01-18

**Authors:** Alistair T Pagnamenta, Rauan Kaiyrzhanov, Yaqun Zou, Sahar I Da'as, Reza Maroofian, Sandra Donkervoort, Natalia Dominik, Marlen Lauffer, Matteo P Ferla, Andrea Orioli, Adam Giess, Arianna Tucci, Christian Beetz, Maryam Sedghi, Behnaz Ansari, Rita Barresi, Keivan Basiri, Andrea Cortese, Greg Elgar, Miguel A Fernandez-Garcia, Janice Yip, A Reghan Foley, Nicholas Gutowski, Heinz Jungbluth, Saskia Lassche, Tim Lavin, Carlo Marcelis, Peter Marks, Chiara Marini-Bettolo, Livija Medne, Ali-Reza Moslemi, Anna Sarkozy, Mary M Reilly, Francesco Muntoni, Francisca Millan, Colleen C Muraresku, Anna C Need, Andrea H Nemeth, Sarah B Neuhaus, Fiona Norwood, Marie O'Donnell, Mary O’Driscoll, Julia Rankin, Sabrina W Yum, Zarazuela Zolkipli-Cunningham, Isabell Brusius, Gilbert Wunderlich, Genomics England Research Consortium, John C Ambrose, Prabhu Arumugam, Emma L Baple, Marta Bleda, Freya Boardman-Pretty, Jeanne M Boissiere, Christopher R Boustred, Helen Brittain, Mark J Caulfield, Georgia C Chan, Clare E H Craig, Louise C Daugherty, Anna de Burca, Andrew Devereau, Greg Elgar, Rebecca E Foulger, Tom Fowler, Pedro Furió-Tarí, Adam Giess, Joanne M Hackett, Dina Halai, Angela Hamblin, Shirley Henderson, James E Holman, Tim J P Hubbard, Kristina ibáñez, Rob Jackson, Louise J Jones, Dalia Kasperaviciute, Melis Kayikci, Athanasios Kousathanas, Lea Lahnstein, Kay Lawson, Sarah E A Leigh, Ivonne U S Leong, Javier F Lopez, Fiona Maleady-Crowe, Joanne Mason, Ellen M McDonagh, Loukas Moutsianas, Michael Mueller, Nirupa Murugaesu, Anna C Need, Peter O’Donovan, Chris A Odhams, Andrea Orioli, Christine Patch, Mariana Buongermino Pereira, Daniel Perez-Gil, Dimitris Polychronopoulos, John Pullinger, Tahrima Rahim, Augusto Rendon, Pablo Riesgo-Ferreiro, Tim Rogers, Mina Ryten, Kevin Savage, Kushmita Sawant, Richard H Scott, Afshan Siddiq, Alexander Sieghart, Damian Smedley, Katherine R Smith, Samuel C Smith, Alona Sosinsky, William Spooner, Helen E Stevens, Alexander Stuckey, Razvan Sultana, Mélanie Tanguy, Ellen R A Thomas, Simon R Thompson, Carolyn Tregidgo, Arianna Tucci, Emma Walsh, Sarah A Watters, Matthew J Welland, Eleanor Williams, Katarzyna Witkowska, Suzanne M Wood, Magdalena Zarowiecki, Mert Karakaya, Brunhilde Wirth, Khalid A Fakhro, Homa Tajsharghi, Carsten G Bönnemann, Jenny C Taylor, Henry Houlden

**Affiliations:** 1 NIHR Biomedical Research Centre, Wellcome Centre for Human Genetics, University of Oxford, Oxford, UK; 2 Department of Neuromuscular Disorders, UCL Queen Square Institute of Neurology, London, UK; 3 Neuromuscular and Neurogenetic Disorders of Childhood Section, NINDS, National Institutes of Health, Bethesda, MD, USA; 4 Department of Human Genetics, Sidra Medicine, Doha, Qatar; 5 Institute of Human Genetics, Center for Molecular Medicine Cologne (CMMC), Institute of Genetics, and Center for Rare Diseases Cologne, University of Cologne, Cologne, Germany; 6 William Harvey Research Institute, Queen Mary University of London, London, UK; 7 Genomics England, London, UK; 8 Centogene AG, Rostock, Germany; 9 Medical Genetics Laboratory, Alzahra University Hospital, Isfahan University of Medical Sciences, Isfahan, Iran; 10 Department of Neurology, Faculty of Medicine, Isfahan University of Medical Sciences, Isfahan, Iran; 11 The John Walton Muscular Dystrophy Research Centre, Institute of Genetic Medicine, Newcastle University, Newcastle, UK; 12 Newcastle upon Tyne Hospitals NHS Foundation Trust, Newcastle, UK; 13 Department of Paediatric Neurology – Neuromuscular Service, Evelina Children's Hospital, Guy's & St Thomas' NHS Foundation Trust, London, UK; 14 Department of Neurology, Royal Devon and Exeter NHS Trust, Exeter, UK; 15 Randall Division of Cell and Molecular Biophysics Muscle Signalling Section, King's College London, London, UK; 16 Department of Basic and Clinical Neuroscience, Institute of Psychiatry, Psychology and Neuroscience, King's College London, London, UK; 17 Donders Institute for Brain, Cognition and Behaviour, Radboud University Medical Centre, Nijmegen, The Netherlands; 18 Department of Neurology, Salford Royal NHS Foundation Trust, Manchester, UK; 19 Department of Genetics, Radboud University Medical Centre, Nijmegen, The Netherlands; 20 West Midlands Regional Clinical Genetics Service and Birmingham Health Partners, Birmingham Women’s and Children’s Hospital NHS Foundation Trust, Birmingham, UK; 21 Divisions of Neurology and Human Genetics, Children's Hospital of Philadelphia, Philadelphia, PA, USA; 22 Department of Pathology, University of Gothenburg, Sahlgrenska University Hospital, Sweden; 23 The Dubowitz Neuromuscular Centre, NIHR Great Ormond Street Hospital Biomedical Research Centre, UCL Great Ormond Street Institute of Child Health, and Great Ormond Street Hospital Trust, London, UK; 24 GeneDx, Gaithersburg, 20877 MD, USA; 25 Mitochondrial Medicine Frontier Program, Division of Human Genetics, Children's Hospital of Philadelphia, PA, USA; 26 Nuffield Department of Clinical Neurosciences, University of Oxford, Oxford, UK; 27 Oxford Centre for Genomic Medicine, Oxford University Hospitals NHS Trust, Oxford, UK; 28 Department of Neurology, King’s College Hospital, London, UK; 29 Peninsula Clinical Genetics Service, Royal Devon and Exeter NHS Trust, Exeter, UK; 30 Division of Pediatric Neurology, The Children's Hospital of Philadelphia, Perelman School of Medicine at the University of Pennsylvania, Philadelphia, PA, USA; 31 Department of Pediatrics, Perelman School of Medicine, Philadelphia, PA, USA; 32 Department of Neurology, Center for Rare Diseases Cologne, University Hospital Cologne, Cologne, Germany; 33 College of Health and Life Sciences, Hamad Bin Khalifa University, Doha, Qatar; 34 Department of Genetic Medicine, Weill Cornell Medical College, Doha, Qatar; 35 School of Health Science, Division Biomedicine and Translational Medicine, University of Skovde, Sweden

**Keywords:** hereditary motor and sensory neuropathies, nerve conduction studies, EMG, genetics: neuropathy, whole-genome sequencing

## Abstract

The extracellular matrix comprises a network of macromolecules such as collagens, proteoglycans and glycoproteins. *VWA1* (von Willebrand factor A domain containing 1) encodes a component of the extracellular matrix that interacts with perlecan/collagen VI, appears to be involved in stabilizing extracellular matrix structures, and demonstrates high expression levels in tibial nerve. *Vwa1*-deficient mice manifest with abnormal peripheral nerve structure/function; however, *VWA1* variants have not previously been associated with human disease. By interrogating the genome sequences of 74 180 individuals from the 100K Genomes Project in combination with international gene-matching efforts and targeted sequencing, we identified 17 individuals from 15 families with an autosomal-recessive, non-length dependent, hereditary motor neuropathy and rare biallelic variants in *VWA1*. A single disease-associated allele p.(G25Rfs*74), a 10-bp repeat expansion, was observed in 14/15 families and was homozygous in 10/15. Given an allele frequency in European populations approaching 1/1000, the seven unrelated homozygote individuals ascertained from the 100K Genomes Project represents a substantial enrichment above expected. Haplotype analysis identified a shared 220 kb region suggesting that this founder mutation arose >7000 years ago. A wide age-range of patients (6–83 years) helped delineate the clinical phenotype over time. The commonest disease presentation in the cohort was an early-onset (mean 2.0 ± 1.4 years) non-length-dependent axonal hereditary motor neuropathy, confirmed on electrophysiology, which will have to be differentiated from other predominantly or pure motor neuropathies and neuronopathies. Because of slow disease progression, ambulation was largely preserved. Neurophysiology, muscle histopathology, and muscle MRI findings typically revealed clear neurogenic changes with single isolated cases displaying additional myopathic process. We speculate that a few findings of myopathic changes might be secondary to chronic denervation rather than indicating an additional myopathic disease process. Duplex reverse transcription polymerase chain reaction and immunoblotting using patient fibroblasts revealed that the founder allele results in partial nonsense mediated decay and an absence of detectable protein. CRISPR and morpholino *vwa1* modelling in zebrafish demonstrated reductions in motor neuron axonal growth, synaptic formation in the skeletal muscles and locomotive behaviour. In summary, we estimate that biallelic variants in *VWA1* may be responsible for up to 1% of unexplained hereditary motor neuropathy cases in Europeans. The detailed clinical characterization provided here will facilitate targeted testing on suitable patient cohorts. This novel disease gene may have previously evaded detection because of high GC content, consequential low coverage and computational difficulties associated with robustly detecting repeat-expansions. Reviewing previously unsolved exomes using lower QC filters may generate further diagnoses.

## Teaser

In a series of 17 individuals with neuropathy and rare biallelic variants in *VWA1*, Pagnamenta *et al.* identify a single frameshift present in the majority of patients. Haplotype analysis suggests that this founder mutation, which is likely to have evaded detection due to its high GC content, may have arisen >7000 years ago.

See Arribat (doi.10.1093/brain/awaa464) for a scientific commentary on this article.

## Introduction

The extracellular matrix (ECM) is a structural and regulatory network of glycoproteins and other macromolecules that plays an important role in connective tissue rich structures such as bone, tendon and skin. The ECM is also important for development and maintenance of the peripheral nervous system including nerve, the neuromuscular junction and muscle. The composition of the ECM varies for different tissues and is highly dynamic. Well-known disease-associated ECM constituents within the peripheral nervous system include perlecan, laminins, collagen Q, collagen XII and collagen VI, which are involved in various aspects of muscle, nerve and neuromuscular junction function and integrity.

In humans, *VWA1* (von Willebrand factor A domain containing 1) encodes a 445 amino acid ECM protein that is also referred to as von Willebrand factor A domain related protein (WARP). Two fibronectin type III repeats are situated downstream of the von Willebrand factor A (VWFA) domain. Reporter gene assays indicated that *Vwa1* is expressed not only in cartilage but also in the basement membrane structures of the peripheral nervous system (Allen *et al.*, 2008). The WARP protein is thought to interact with perlecan and also type VI collagen. In WARP-deficient mice there is compromised function of peripheral nerves and collagen VI is reduced in regions of the peripheral nerve ECM (Allen *et al.*, 2009). Transmission electron microscopy of sciatic nerve tissues taken from the WARP-deficient mice revealed an unusual partial fusion of the basement membranes of neighbouring axons (Allen *et al.*, 2009). In humans, pathogenic variants in collagen 6 (*COL6A1–3*) genes cause COL-6-related dystrophies (COL6-RDs) ranging from the severe Ullrich congenital muscular dystrophy (MIM #254090), to intermediate phenotypes and Bethlem muscular dystrophy at the milder end of the spectrum (MIM #158810).

Human gene expression data (GTEx Consortium, 2015) indicate that *VWA1* shows highest expression levels in tibial nerve (Supplementary Fig. 1A). Other well-known disease genes *PMP22* (MIM *601097) and *MPZ* (MIM *159440) that are linked to subtypes of Charcot-Marie-Tooth (CMT) disease also demonstrate high relative expression levels in tibial nerve (Supplementary Fig. 1B and C). Thus, although to date there have not been any human disorders associated with this gene, *VWA1* represents an interesting candidate for peripheral nerve related phenotypes.

In this study, a combination of genome, exome and targeted sequencing approaches was used in tandem with international gene-matching and autozygosity mapping to identify 15 families with biallelic variants in *VWA1.* RNA analysis and a number of *in silico* tools were used to predict the likely consequences of the variants uncovered whilst Western blotting assessed mutational effects at the protein level. Detailed clinical information for this cohort is presented and for a subset of families, availability of genome sequencing data facilitated a high-resolution haplotype analysis for individuals homozygous for the primary disease-associated allele. Finally, the effect of defective Vwa1 on axonal development and synapse formation was assessed in zebrafish.

## Materials and methods

### Analysis of genome sequence data within the GEL research environment

For Families 1–10, genome sequencing was performed as part of the 100K Genomes Project (100KGP), a national genome sequencing initiative (Turnbull *et al.*, 2018; Turro *et al.*, 2020). For the majority of cases, sequencing was performed using 150-bp paired-reads on a HiSeqX instrument (Illumina). In this study, we searched data from release v8 (2019-11-28) of the 100KGP, specifically looking for rare biallelic variants in *VWA1*. Multiple filtering strategies were used, as described in Supplementary material. Of the 10 families identified below, five patients had been sequenced as singletons, four as parent-child trios and one as a mother-child duo. Genomics England has approval from the HRA Committee East of England, Cambridge South (REC: 14/EE/1112).

### Haplotype analysis in seven individuals homozygous for p.G25Rfs*74

Joint variant calling was performed across all seven individuals homozygous for the p.G25Rfs*74 variant (Supplementary material). For all high-confidence SNVs with a minor allele frequency of >1%, we plotted information content (reciprocal of 1000 Genomes project allele frequency) versus genomic position where single nucleotide polymorphisms (SNPs) were shared homozygous in 7/7. For some individuals the ancestral haplotype extended further and so review of the multi-sample VCF and the BAM files was performed using IGV to delineate precise region lengths. The ancestral haplotype block sizes were used to estimate the age of the p.G25Rfs*74 variant, as described previously (Gandolfo *et al.*, 2014). For comparison, we performed an identical analysis for 10 unrelated ataxia patients from the 100KGP, homozygous for the intronic AAGGG repeat expansion in *RFC1* (Cortese *et al.*, 2019)*.* These individuals had been detected in using ExpansionHunter v2.5.5.

### Structural analysis of VWA1

Structural analysis of VWA1 was calculated with PyRosetta (Chaudhury *et al.*, 2010) and results are presented via a web-based application (Ferla *et al.*, 2020) that promotes the sharing of 3D macromolecular visualizations.

### Identification of families with *VWA1* variants through international gene-matching efforts

Identification of additional families with biallelic variants in *VWA1* was performed by using GeneMatcher (Sobreira *et al.*, 2015) and a network of established collaborators. For Family 11, exome sequencing was performed as a trio at GeneDx (Gaithersburg, MD) as described (Retterer *et al.*, 2016). Variants were scrutinized using Xome Analyzer, a custom-developed analysis tool. The proband in Family 12 was exome sequenced in the Nijmegen diagnostic laboratory as a singleton, in collaboration with BGI Europe. Sequencing was on an Illumina HiSeq instrument after exome enrichment with the Agilent SureSelectXT Human All Exon 50Mb Kit.

### Autozygosity analysis in an extended kindred

For Family 13, exome enrichment was performed on the proband and his uncle using the SureSelectXT Human All Exon Kit V6 (Agilent Technologies). Sequencing was on an Illumina NovaSeq instrument as paired-end 2 × 150-bp reads with ∼50× coverage. Genetic variants were identified using the GATK suite v4.0.4.0 and the multi-sample vcf was uploaded to www.homozygositymapper.org for analysis, with the requirement for genetic homogeneity. Variants were also analysed using Ingenuity Variant Analysis™ (Qiagen, Redwood City). Given the known consanguinity in this family, we focused on rare homozygous variants present in regions of autozygosity shared between the proband and the uncle. A more detailed description of variant filtering is available in the Supplementary material.

### Targeted sequencing of replication cohort

Sanger sequencing was performed in two independent cohorts; 374 unrelated patients from UK with a range of complex peripheral neuropathies and 967 unrelated patients suspected of spinal muscular atrophy from Cologne. The latter cohort was selected based on negative results upon *SMN1* testing, with patients >2 years of age when samples were received. Polymerase chain reaction (PCR) was used to amplify exon 1 of *VWA1* using ∼85–100 ng of template DNA. Because of the high GC content, Q5 high GC enhancer (BioLabs) or DMSO (Applichem) was added for efficient amplification. Enzymatic clean-up followed with a mixture of FastAP^TM^ Thermosensitive Alkaline Phosphatase and ExoI (Thermo Scientific or NEB). Samples were either sent to SourceBioscience, Eurofins, Microsynth or sequenced in house using BigDye^®^ (Applied Biosystems or Life Technologies). Following a Sephadex^TM^ G-50 purification step, sequences were read by a 3500/3730 DNA Analyzer and analysed using Sequencher (GeneCodes) or SeqPilot (JSI Medical Systems).

### Clinical data collection

A detailed clinical *pro forma* was circulated to all clinicians from February 2020 onwards, along with requests for neurophysiology data, muscle histology results, muscle MRI data and any photos/videos where consent was forthcoming. Written informed consent for genetic testing and photo materials were obtained from the patients, parents or legal guardians. The study was conducted in accordance with the Declaration of Helsinki and approved by the relevant institutional review boards.

### Analysis of *VWA1* transcript levels

Following informed consent (12-N-0095, NIH, NINDS IRB), a skin biopsy was performed on the proband from Family 11 and a dermal fibroblast cell line was established using a standard protocol. Total RNA was isolated with the RNeasy^®^ mini Kit (Qiagen) and reverse transcribed into cDNA with SuperScript^TM^ IV reverse transcriptase (ThermoFisher Scientific). *VWA1* transcript levels in patient fibroblasts were compared to that in control fibroblasts using duplex PCR, with primers in *VWA1* (GAGCGAGCGAGCGAGTTG, GTCCAGCAGGAACATCAGGT) and control primers targeting *ACTB*. To determine if the 10-bp insertion causes nonsense-mediated decay, dermal fibroblasts were treated with 0.2 mg/ml cycloheximide (CHX; Sigma) for 19 h.

### VWA1 abundance in cultured dermal fibroblasts

Confluent cultures of dermal fibroblasts from the patient in Family 11 and a healthy control were grown in serum free medium for 40 h. Conditioned medium and cell layer were then collected separately. Protein from the conditioned medium was precipitated with cold ethanol and dissolved in 2× LDS buffer (ThermoFisher Scientific). The protein lysate from the cell layer was prepared with 4 M guanidine hydrochloride (GuHCl), as described (Allen *et al.*, 2008). Aliquots of the above lysates were analysed by western blot using 4–12% SDS-PAGE under reducing conditions followed by transfer to PVDF membrane (Millipore) and incubation with sheep antibody against VWA1 (Allen *et al.*, 2008). Protein lysates prepared from mouse nerve tissue and mouse nerve primary culture were also loaded as positive controls. The same membrane was also blotted with antibodies against fibronectin and tubulin as loading controls.

### Zebrafish *vwa1* modelling

Zebrafish (*Danio rerio*) wild-type and transgenic (Tg:olig2:dsRed) adults were maintained in standard conditions under approved protocol by the local Animal Care (QU-IACUC 26-2/2018-REN1). Precision gene editing and transient knockdown approaches targeted ENSDARG00000075468, the zebrafish orthologue of the human gene *VWA1*. Zebrafish *vwa1*-specific guide RNAs were TGTAGAAGAGGTTAAACTGG and CAGAACACAGAAACACGGTG. Morpholinos (MOs) targeting the three protein-coding transcripts were MO: 5′ACATGACCAAAACGACTCACCTGAA3′-Blue and a Standard Control MO: 5′CCTCTTACCTCAGTTACAATTTATA3′ (Gene Tools). Injections were performed as described previously, with a final dose of 0.5 mM (Da'as *et al.*, 2020).

### Motor neuron length measurements

Phenotypic assessment and imaging was performed on the olig2-labelled elongated axons. Larvae were immobilized in 3% methylcellulose (Sigma, M0387) for imaging. The motor axon length was measured for 6–10 axons per larva using DanioScope software (Noldus).

### Immunofluorescence of motor neurons, phalloidin and neuromuscular junction imaging

Immunofluorescence was performed as described (Sufian *et al.*, 2019). Fixed zebrafish groups were incubated overnight at 4°C in mouse monoclonal anti-znp1 or anti-zn8 (Developmental Studies Hybridoma Bank) antibodies then incubated in the secondary antibody, Alexa Fluor^®^ 488 goat anti-mouse IgG overnight at 4°C. Phalloidin and alpha-bungarotoxin staining was performed as described (Bailey *et al.*, 2019). Samples were co-stained in 1/20 phalloidin-488, which was added to the alpha-bungarotoxin solution in PBS-2% Triton^TM^ X-100 overnight at 4°C.

### Cartilage Alcian blue staining

Cartilage was stained via a modified version of established protocols (Delbaere *et al.*, 2019; Gebuijs *et al.*, 2019). Larvae were fixed for 6 h in 4% paraformaldehyde. Specimens were bleached for optical clarity in 1% hydrogen peroxide, 1% potassium hydroxide and 0.2% Triton for 1 h. Staining with Alcian blue was performed for 3 h and this was followed by a clearing step in acidic ethanol (5% hydrochloric acid, 70% ethanol) for 10 min then transferred to pure glycerol for imaging.

### Zebrafish locomotor behaviour measurements

Tail flicking activity was assessed as described (Basnet *et al.*, 2017). Briefly, the spontaneous tail coiling was evaluated by acquiring 20 s interval video recording for the embryos at 24 hours post fertilization (hpf). The video was obtained at 60 frames per second setting using Imaging Source camera. Locomotion activity of embryos was evaluated by analysing the acquired videos using DanioScope software (version 1.1, Noldus, The Netherlands). Each embryo was selected by drawing a separate arena around its chorion to detect the movement of the tail. The locomotion activity was measured for 10–20 embryos, and the results were compared to the control groups.

Zebrafish larvae locomotor activity was monitored at 120 hpf using an automated Video-Track system (Noldus, Ethovision XT), as described (Basnet *et al.*, 2019). Larvae were placed individually in a six-well plate. Larval swimming behaviour was monitored in response to dark-to-light transitions. The locomotor behaviour was monitored, and the collected data were analysed using Ethovision XT software.

### Data availability

Information about how to access data from the 100KGP by joining a Genomics England Clinical Interpretation Partnership (GeCIP) is available online (www.genomicsengland.co.uk/join-a-gecip-domain).

## Results

### Biallelic *VWA1* variants in the 100K Genomes project

**Figure 1:**
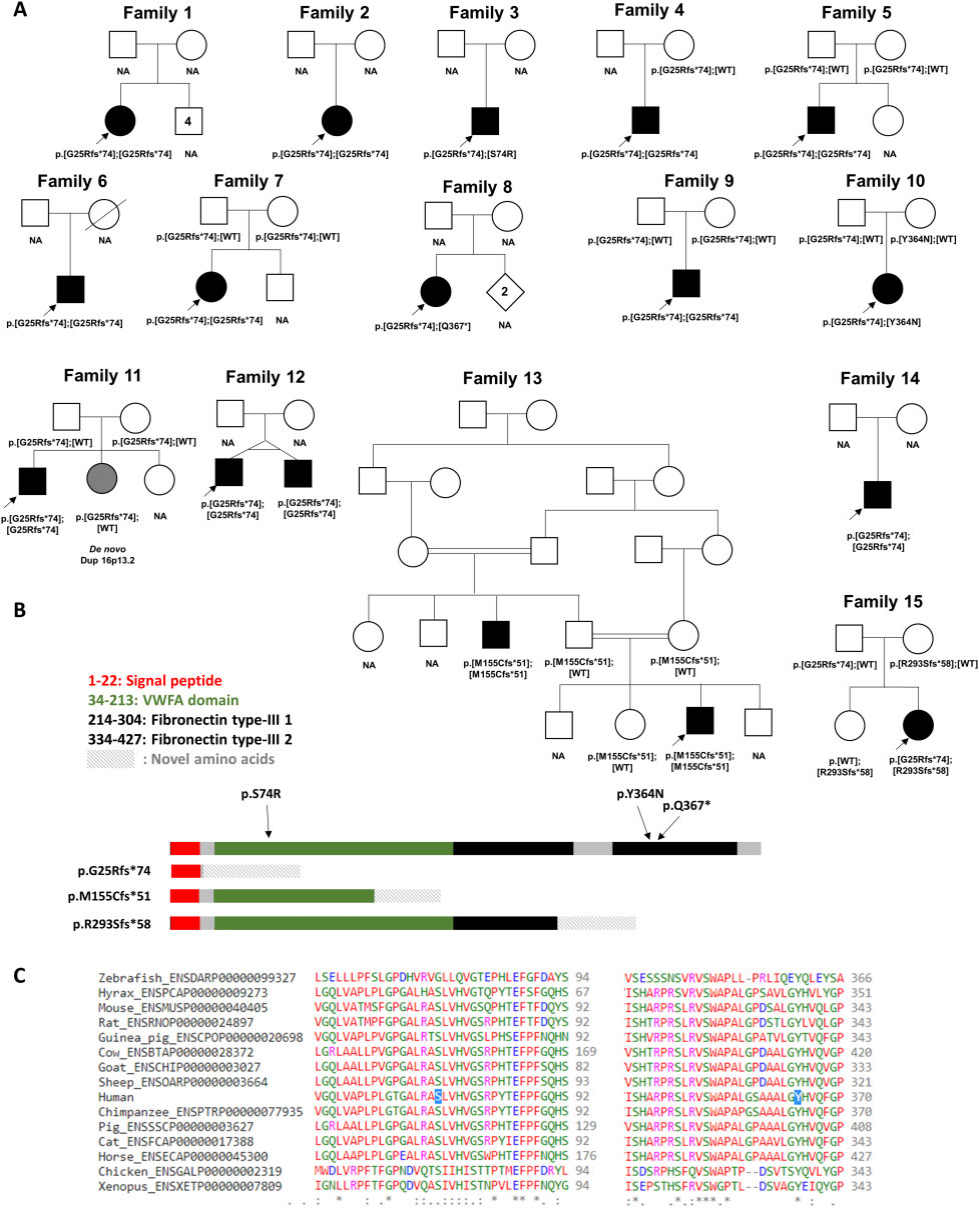
**Pedigrees, variant localization and evolutionary conservation of missense changes in *VWA1.*** (**A**) Pedigrees of the 15 families described here with predicted protein consequences of variants and the segregations pattern, where known. NA = DNA not available for testing. Filled symbols indicate early onset motor axonal neuropathy. Grey shading in Family 11 indicates a neurological presentation consistent with the observed duplication of 16p13.2. Genotype in younger twin in Family 12 was inferred because of monozygosity. (**B**) Schematic diagram showing the position of the variants identified in this study in relation to the protein domains in VWA1. The VWFA domain is shown in green whilst the two fibronectin type-III domains are shown in black. Figure is based on coordinates as listed in entry Q6PCB0 of the UniProt database (www.uniprot.org). (**C**) Evolutionary conservation of amino-acids in VWA1 orthologues at p.Ser74 and p.Asn364, the two sites where missense changes were identified.

We searched data from the 100KGP (Turnbull *et al.*, 2018) for families where affected individuals harboured biallelic variants in *VWA1*. Ten families of interest were uncovered, all of which harboured a 10-bp insertion (c.62_71dup10; p.G25Rfs*74, NM_022834.5). In seven families, this variant was observed in the homozygous state in affected individuals, whereas in the other three families the p.G25Rfs*74 allele was heterozygous alongside a rare heterozygous SNV (Fig. 1A). Two of these SNVs predict missense alterations (p.S74R and p.Y364N) in functional protein domains involving conserved residues (Fig. 1B and C) whilst the other predicts a stop-gain (p.Q367*). The p.Ser74 residue has previously been shown to undergo phosphorylation (Tagliabracci *et al.*, 2015). Structural analysis suggests that p.S74R and p.Y364N are both likely to destabilize VWA1, with ΔΔG values of +24 and +10 kcal/mol, respectively (interactive view: https://michelanglo.sgc.ox.ac.uk/r/vwa1), a higher predicted change in Gibbs free energy than obtained for the six homozygous missense variants observed in gnomAD. Other *in silico* predictions for these variants are available in Supplementary Table 1.

For Families 3 and 8, affected individuals were genome sequenced as singletons. As both individuals are >70 years old, parental samples were not available to establish the phase of the variants. For Family 3, allele-specific PCR was used to confirm that the p.G25Rfs*74 and p.S74R variants lay *in trans* (Supplementary Fig. 2). For Family 8, the two variants lay 3.75 kb apart. Nanopore sequencing on the affected individual, resulted in a genome-wide coverage of ∼13× and a mean aligned read-length of 16 017 bp. These data suggested that p.G25Rfs*74 and p.Q367* were oriented *in trans* (Supplementary Fig. 3). In Family 10, compound-heterozygosity of p.G25Rfs*74 and p.Y364N was already established as both parents had been sequenced in parallel. These 10 unrelated patients from the 100KGP had been recruited under four related diagnostic categories, the most common being CMT disease and paediatric motor neuronopathies, where 0.85/0.86% cases were identified with biallelic *VWA1* variants (Table 1).

**Table 1:** Incidence of *VWA1* cases with biallelic variants identified in the 100K GP across different diagnostic categories

Disease subgroup	Specific disease	Biallelic *VWA1* cases	**Participant count** ^a^	Frequency
Motor and sensory disorders of the peripheral nervous system	Charcot-Marie-Tooth disease	6^b^	708	0.85%^c^
	Paediatric motor neuronopathies	1	116	0.86%
Neuromuscular disorders	Congenital myopathy	2	479	0.42%
	Limb girdle muscular dystrophy	1	253	0.40%
Above diseases combined		10	1556	0.64%
All 100KGP participants with specific disease		10	40 088	0.02%
All 100KGP participants^d^		10	74 180	0.01%

^a^ Numbers based on CohortBrowser v8 (28 November 2019).

^b^ Contains one case with p.[G25Rfs*74];[S74R] phased by allele-specific PCR, one case with p.[G25Rfs*74];[Q367*] phased by Nanopore sequencing and one case with p.[G25Rfs*74];[Y364N] phased by inheritance.

^c^ This frequency rises to 1.12% (6/535) if the denominator is recalculated considering only the proband in each family and removing individuals where ethnicity is reported as Black, Asian, Chinese or ‘other’.

^d^ Includes unaffected family members.

### Haplotype analysis suggests p.G25Rfs*74 is an ancient European founder mutation

**Figure 2:**
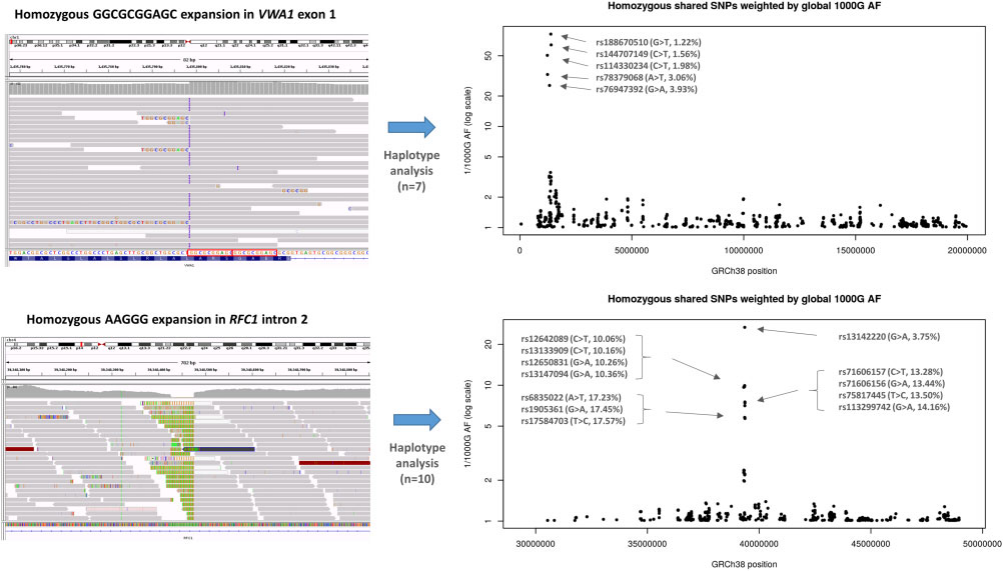
**Haplotype analysis performed on individuals with homozygous repeat expansions in *VWA1* (*top*) compared to similar results for *RFC1* (*bottom*).** Shared homozygous SNPs are plotted for 20 Mb segments of chromosome 1 and 4. In both cases, the most significant regions detected span the *VWA1* and *RFC1* loci where several consecutive SNPs are homozygous in 7/7 or 10/10 individuals respectively. For the *VWA1* locus, the five shared SNPs labelled with rsIDs are highly informative, with global 1000 Genomes project allele frequencies <5% (1000G AF). The reciprocal of the allele frequency is plotted on the *y*-axis to give an idea of the information content for each of the shared SNPs. Within the *VWA1* haplotype block we also observed a rare SNV in the 5′-UTR of *ANKRD65* (rs758603246), which is heterozygous in 3/7 individuals. The variants appear to be in complete linkage disequilibrium as in the 100KGP all individuals with rs758603246 also have p.G25Rfs*74. Our interpretation is that rs758603246 is a more recent mutation and so is observed only on a subset of p.G25Rfs*74-containing haplotypes.

Expansions of repetitive stretches of DNA are considered to be one of the more recurrent forms of genetic variation. However, the 10-bp repeat in exon 1 of *VWA1* only has two copies in the reference and the expansion to three copies (p.G25Rfs*74) appears to be a relatively stable founder mutation. Of the 89/140 632 expanded (3 × 10 bp) alleles seen in gnomAD v3, all are in a heterozygous state and the majority (58/89) are in individuals with non-Finnish European ancestry. Haplotype analysis performed on the seven homozygous individuals where genome sequence data were available identified a shared haplotype of 220.4 kb that extends mainly in the distal direction. For the most informative SNPs on this haplotype, the alternate alleles (rs114330234-rs78379068-rs76947392-rs188670510-rs144707149; T-T-A-T-T) are observed at 1.2–3.9% in the 1000 Genomes project (Fig. 2). The three compound heterozygous individuals (Families 3, 8 and 10) are also heterozygous for this set of alleles. The small size of this shared ancestral haplotype makes it unlikely that the *VWA1* founder mutation is from a recent common ancestor (Speed and Balding, 2015). Further refinement of the founder haplotype showed that for some patients the region extended up to 471.5 kb (Supplementary Table 2). Using a previously described method (Gandolfo *et al.*, 2014), we estimated this mutation to be 311 generations old (95% confidence interval: 107–975; Supplementary Table 3). Assuming an average of 25 years per generation, this equates to 7775 years (Supplementary Table 4). These haplotype data may be useful in prioritizing European neuropathy patients for targeted *VWA1* sequencing, for instance rs78379068 is genotyped on Illumina’s Infinium Global Diversity Array. For comparison, we assessed 100KGP data for 10 individuals homozygous for the AAGGG insertion in *RFC1*, also a known disease-causing repeat expansion and present in Europeans at an allele frequency of ∼0.7% (Cortese *et al.*, 2019). An ancestral haplotype of 58.2 kb was seen across all individuals (Fig. 2), suggesting that this variant is likely to be older than the *VWA1* founder mutation.

### Families detected via exome sequencing, international collaboration and autozygosity analysis

A further five affected individuals from three independent families were identified (Fig. 1A) by exome sequencing. Similar to Families 1–10, Families 11 and 12 are both small outbred kindreds of European ethnicity where affected individuals harboured the homozygous p.G25Rfs*74 founder variant. In contrast, Family 13 is of Afghan origin and the only family in which consanguinity was documented. Retrospective homozygosity mapping detected a 1.5 Mb region spanning *VWA1* (chr1:137825–1646565 GRCh38/hg38) comprising 11 consecutive homozygous SNPs as the joint second highest scoring region (Supplementary Fig. 4 and Supplementary Table 5). The only larger regions detected were on chromosomes 3 and 7. In the proband’s exome, 712 rare (0.5% or less) deleterious variants were identified, of which 80 were called homozygous. Filtering these variants for those in shared autozygous regions identified c.462delC; p.M155Cfs51 in *VWA1* as the only candidate variant.

### Genetic replication via targeted sequencing

In an attempt to replicate the results obtained from genome/exome sequencing, 1341 DNA samples from two independent cohorts of patients (374 from UK, 967 from Germany) with unsolved neuropathies were tested for the p.G25Rfs*74 founder mutation by screening exon 1 using a Sanger sequencing approach. This yielded three additional diagnoses: (i) an 83-year-old male with suspected non-length dependent hereditary motor neuropathy who harboured the homozygous p.G25Rfs*74 variant; (ii) a 24-year-old female with distal axonal motor neuropathy who was compound heterozygous for p.G25Rfs*74 and p.R293Sfs*58 (Fig. 1A) and (iii) a compound heterozygous individual (p.G25Rfs*74 and p.R32*) who is described elsewhere (Deschauer *et al.*, 2020) and so detailed phenotypic information is not presented.

### Phenotype range associated with biallelic *VWA1* variants

Clinical data were assembled from 17 affected individuals across 15 independent families, all shown to carry biallelic *VWA1* variants (Table 2, Supplementary Table 6 and Supplementary material, case reports). Videos are available for affected probands in Families 1, 3, 8, 10–13 and 15 (Supplementary Videos 1–8) and additional clinical images are shown for the proband from Family 13 (Supplementary Fig. 5). Although there was a wide age range (6–83 years), children made up 35% of this series. Positive family history was reported only in Family 13, while the rest of the cases were sporadic. The common disease presentation in the cohort was an early-onset slowly progressive non-length dependent predominantly axonal motor neuropathy, often with early distal weakness and foot deformities progressing over the years to involve proximal muscle groups.

**Table 2:** Summary of clinical features in the VWA1 cohort

Clinical feature/demographic	
Number of individuals	*n *=* *17
Ethnicity	
White British	6 (35%)
Mixed British	3 (18%)
Caucasian non-British	4 (23%)
Afghan	2 (12%)
Unknown	2 (12%)
Gender	11 males/6 females
Family history (of NMDs)	3 (18%)
Consanguinity	2 (12%)
Current age	Median 42
Current age <11 years	6 (35%)
Age of symptom recognition, years	Mean 2.0 ± 1.4
Disease duration	Median 36.5
Age at examination	Median 37.5
Age of independent walking	Mean 1.6 ± 0.8 (12)
Slow disease progression	17 (100%)
Joint flexion contractures	9 (53%)
Spinal deformities (Scoliosis, lumbar hyperlordosis)	8 (47%)
Foot deformities	15 (88%)
Pes cavus	11 (65%)
Talipes equinovarus	6 (35%)
Foot surgery	3 (18%)
Myalgia	7 (41%)
Frequent falls	6 (35%)
Forward stood posture	6 (35%)
Loss of independent ambulation	3 (18%)
Tongue fasciculations or atrophy	2 (12%)
Scapular winging	2 (12%)
Lower limb weakness	
Weakness in the proximal muscle groups	13/16 (81%)
Weakness in the distal muscle groups	15/16 (94%)
Only distal weakness	3/16 (19%)
Only proximal weakness	1/16 (6%)
Simultaneous proximal and distal weakness	12/16 (75%)
Proximal > distal weakness	5/12 (42%)
Distal > proximal weakness	4/12 (33%)
Proximal = distal weakness	3/12 (25%)
Foot drop	9 (53%)
Impaired toe walking	6/13 (46%)
Lower limb amyotrophy	10 (59%)
Upper limb involvement	11 (65%)
Upper limb weakness	
Proximal muscle groups	10 (59%)
Distal muscle groups	8 (47%)
Impaired sensation	3/15 (20%)
Paraesthesia	3 (18%)
Hypotonia	1 (6%)
Hyporeflexia	8/16 (50%)
Myopathic gait	6 (35%)
Motor axonal neuropathy on NCS	15/15 (100%)
Myogenic changes on EMG	3/12 (25%)

Denominator is 17 unless otherwise stated. NCS = nerve conduction studies; NMDs = neuromuscular disorders.

The mean age of symptom recognition was 2.0 ± 1.4 years with tip-toe walking, foot deformities, Achilles tendon contractures, and recurrent hip and patellar dislocations. Independent walking in the cohort was achieved by the mean age of 1.6 ± 0.8 years and it was remarkably delayed in cases with foot deformities such as severe bilateral equinovarus (18%, 3/17). In two independent cases, the foot deformities and tendon contractures were congenital suggesting antenatal onset of the disease. The aforementioned presenting symptoms in the cohort were typically followed by the development of slowly progressive symmetric distal and/or proximal muscle weakness in the lower limbs. In over half of the affected individuals, weakness spread to the upper limbs over time predominantly affecting the proximal muscles.

Most of the cases (75%) were found to have simultaneous proximal and distal lower limb weakness on neurological follow-up. In almost half of those cases, weakness was more pronounced proximally, and one-quarter had equally weak proximal and distal muscle groups, raising the clinical suspicion of non-length dependent motor axonal neuropathy. Weakness was mild in the upper limbs, and ranged from mild to near-complete paralysis in the proximal and distal lower limbs, irrespective of age.

Bilateral foot drop, impaired toe walking, forward posture, and wasting in the lower limbs were frequently observed signs. Of note, bilateral foot deformity was a very frequent feature revealed in almost 90% of the cases (15/17), ranging from pes cavus (65%, 11/17) to talipes equinovarus (35%, 6/17). Less frequent but present in more than half of the cohort were flexion tendon contractures typically involving foot plantar flexion, knee, and hip flexion but in some cases, it was also present in the major joints of the upper limbs. Scoliosis and lumbar hyperlordosis were present in half of the series. One-third of the cases experienced frequent falls but despite the long disease course (median disease duration 36.5 years), only three members of the cohort (twins from Family 12 and index case from Family 6) had lost independent ambulation. Three isolated cases had an abnormal sensory examination and this was supported by neurophysiological evidence in only one of them showing asymmetrical sural nerve involvement (Family 6). Tendon reflexes were diminished in half of the cohort, and muscle tone was largely reported to be normal along with uniformly negative Babinski sign. Two adult cases (Families 1 and 3) had shown mild tongue fasciculations or atrophy.

Based on the presence of proximal muscle weakness in the upper and lower limbs associated with myalgia, myopathic gait, and scapular winging, several cases were suspected to have additional clinical signs suggestive of myopathy. In six affected individuals, creatine kinase levels were elevated (369–1628 IU/l).

The homozygous carriers of p.(G25Rfs*74) largely showed a comparable pattern of neuromuscular and associated symptoms. Cases with compound heterozygous *VWA1* variants p.(G25Rfs*74); p.(S74R) and p.(G25Rfs*74); p.(Q367*), both >70 years old, as well as the 24-year-old with p.[G25Rfs*74];[R293Sfs*58] seemed to present with a milder phenotype. Overall, the disease progression was very slow or even static in several cases. Isolated cases reported some clinical worsening after the fifth decade of life.

Available nerve conduction studies typically revealed findings consistent with a motor axonal neuropathy which was often interpreted as suggestive of a distal spinal muscular atrophy/hereditary motor neuropathy. Motor nerve action potentials from the lower limb nerves were reduced or undetectable with uniformly preserved conduction velocities. Sensory studies were overall normal. EMG typically showed chronic neurogenic changes without active denervation (10/12). Three EMG studies were reported to suggest myopathic features, albeit for two of them the formal report was unavailable. Table 3 displays the nerve conduction studies and EMG parameters from the formal reports available.

**Table 3:** Summary of neurophysiology results

Family number	Age, years	Median motor		Ulnar motor		Peroneal motor		Tibial motor		Median sensory		Ulnar sensory		Radial sensory		Sural sensory		Peroneal sensory		EMG LL
		Amp mV	CV	Amp mV	CV	Amp mV	CV	Amp mV	CV	**Amp µV**	CV	**Amp** µV	CV	**Amp** µV	CV	**Amp** µV	CV	**Amp** µV	CV	
F1	50	6.8	55.4	ND	ND	ND	ND	0.53	38	10.2	69.3	5.2	64.7	ND	ND	6.7	59.6			+
F4	8	ND	ND	ND	ND	UD	UD	2.2	41	ND	ND	ND	ND	ND	ND	8.6	50	17.7	54	+
F6	40	24.5	56.8	ND	ND	UD	UD	ND	ND	ND	ND	ND	ND	60.1	54.9	7.30	41.7	5.2	36.6	×
F8	70	7.7	59.1	7.3	62.5	3.6	54	1.1	50	21	57.4	10	59.9	ND	ND	33	68.2	18	47.3	+
F11	8	2.7	48	4.6	55	0.5	48	0.8	46	ND	ND	ND	ND	23.9	54	16.3	48			ND
F12^a^	57	7.1	51.6	7.2	55	UD	UD	2.7	33.9	ND	ND	ND	ND	20.5	53.3	6.2	38.4			+
F13^b^	8	5.9	49	4.6	54	0.2	41	2.7	47	18.2	53	15.4	54	ND	ND	7.2	66	11	47	++
F15	24	ND	ND	17.3	66	4.3	44	13.5	48	ND	ND	34	53	ND	ND	19.1	43	ND	ND	+

Nerve conduction study parameters are those from the right limbs. Amp = amplitude; CV = conduction velocity (m/s); LL = lower limbs; ND = not done; UD = undetectable (no response); + = chronic denervation; × = acute denervation; ++ = early recruitment of short duration polyphasic motor unit action potentials and profound myotonic discharges.

^a^ Elder twin.

^b^ Proband.

**Figure 3:**
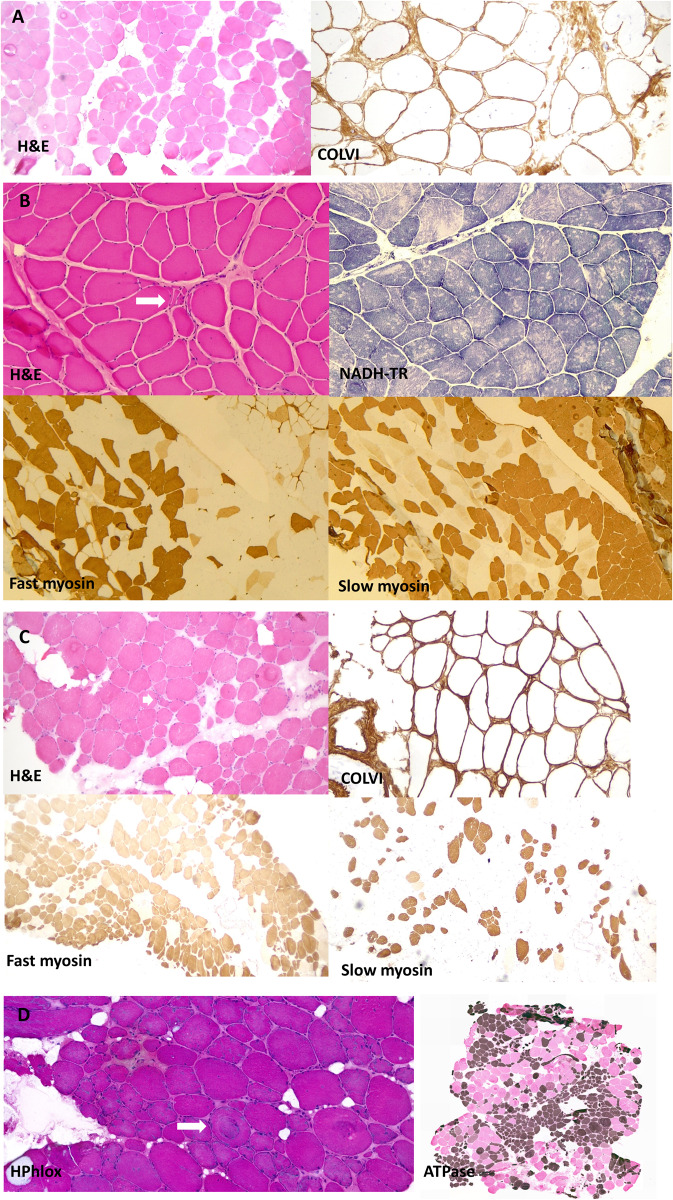
Muscle biopsy images from our patients all homozygous for p.G25Rfs*74. (**A**) Muscle biopsy (quadriceps) from a patient in Family 2 (performed at age 41 years). Images show marked fibre hypertrophy and increased fibrosis with endo and perimysial fatty infiltration. Labelling for collagen VI appears normal. (**B**) Muscle biopsy (right quadriceps) from a Family 4 patient (aged 6 years). Biopsy shows predominantly neuropathic aspects with neurogenic atrophy (arrow) and thickening of the endomysium and perimysium. Fascicular grouping of type 1 fibres implicates a chronic neurogenic process. NADH-TR shows the presence of moth-eaten fibres (non-specific finding). (**C**) Muscle biopsy (quadriceps) from a patient from Family 5 (aged 41 years). Marked variation in fibres size and increased endomysial fibrosis with mostly perimysial fatty infiltration. Rimmed vacuoles are shown with an arrow. There is a large predominance of type 2 fibres. Labelling for collagen VI appears normal. (**D**) Muscle biopsy from the index case in Family 12 (right vastus lateralis, age 52). Myopathic features are present, with marked fibre lobulation and variable degree of whorling of myofibrils (arrow). Endomysial fatty infiltration. Large areas of fibre grouping in this biopsy as well indicates a neurogenic aetiology. ATPase staining was performed at pH 4.2. H&E = haematoxylin and eosin.

Muscle biopsies were performed in seven cases, of which four were available for review (Fig. 3). In 4/7 of these, clear neurogenic changes were reported. In the other three biopsies there was evidence for myopathic features in addition to findings suggestive of an underlying neurogenic process. Where data were available, immunohistochemical labelling for collagen VI appeared normal.

Muscle MRIs were available in four cases, of which three were available for review. This displayed increased signal intensity or fatty replacement predominantly in the vastus lateralis and anterior compartment of the lower legs on T_1_-weighted sequences suggesting chronic denervation (Supplementary Fig. 6). In one affected individual a muscle MRI was interpreted as compatible with a myopathic condition; however, the MRI images from this case were not available for review.

### Transcripts harbouring founder mutation are partially degraded by nonsense-mediated decay in fibroblasts

**Figure 4:**
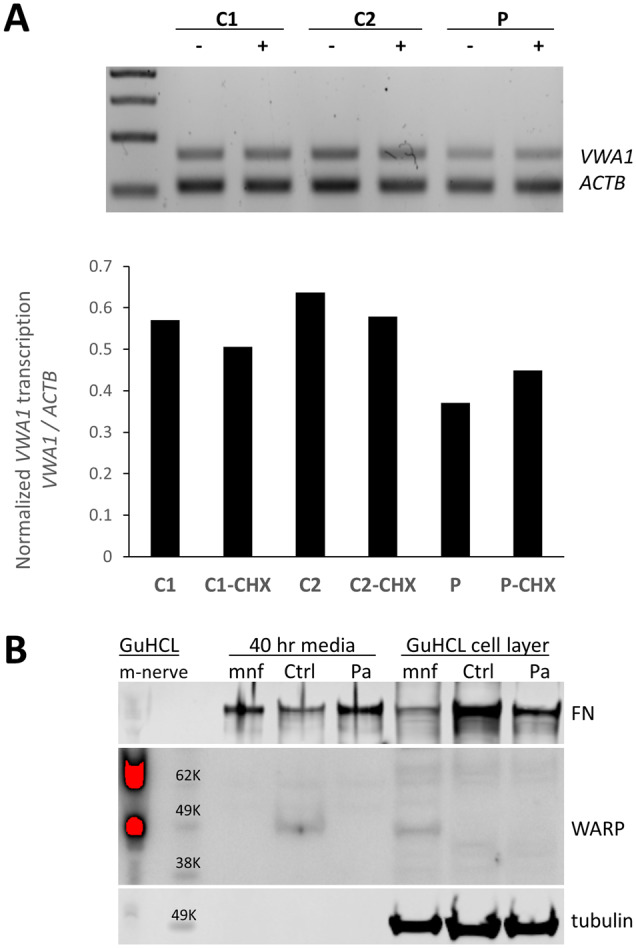
**Consequences of the 10 bp insertion at the transcript and protein level.** (**A**) Duplex PCR suggests a 10 bp insertion results in partial nonsense-mediated decay. RT-PCR products using primers for *VWA1* and *ACTB* using RNA from proband in Family 11 (P) compared to control fibroblasts (C2, C2). The ratio of *VWA1* to *ACTB* was reduced in the patient but was restored when patient fibroblasts were grown in presence of the nonsense-mediated decay inhibitor cycloheximide (CHX). (**B**) Immunoblotting indicated detectable levels of VWA1 (WARP) in human healthy control dermal fibroblasts; although the secreted VWA1 was not able to incorporated into ECM (cell layer), it was detectable in the conditioned medium. In contrast, no detectable VWA1 was seen in the patient’s dermal fibroblast in either the conditioned medium or the cell layer. A high level of VWA1 was detected in mouse sciatic nerve extraction (m-nerve). In the primary culture of mouse sciatic nerve (mnf), the VWA1 was detectable in its cell layer (cytoplasm and ECM) but not in the conditioned medium, indicating the secreted VWA1 was efficiently deposited as ECM. Tubulin and fibronectin (FN) used as loading controls. GuCHL = guanidine hydrochloride extraction.

The major disease-associated insertion allele results in a frameshift and the introduction of a premature stop signal 74 codons downstream. Mutant transcripts would therefore be predicted to be subject to nonsense-mediated decay. To test this possibility, duplex reverse transcription polymerase chain reaction (RT-PCR) was performed using RNA from dermal fibroblasts obtained from the proband in Family 11. *VWA1* transcript levels were reduced compared to two controls. The *VWA1*/*ACTB* ratio was partially restored when patient fibroblasts were grown in presence of cycloheximide (Fig. 4A) suggesting that the 10 bp insertion results in transcripts that are partially degraded by nonsense-mediated decay.

### No detectable VWA1 protein in patient fibroblasts

Detectable levels of VWA1 were seen in dermal fibroblasts from a healthy control. Although secreted VWA1 was not able to incorporate into ECM (cell layer), it was detectable in the conditioned medium. However, there was no detectable VWA1 in either the conditioned medium or cell layer of the patient’s fibroblast (Fig. 4B). A high level of VWA1 was detected in mouse sciatic nerve extraction. In the primary culture of mouse sciatic nerve, VWA1 was detectable in its cell layer (cytoplasm and ECM) but not in the conditioned medium, indicating the secreted VWA1 was able to deposit as ECM efficiently.

### Zebrafish *vwa1* model leads to motor neuron malformations

The zebrafish Vwa1 protein shares 64.7% similarity across a 408 amino acid overlap with human VWA1. Zebrafish were successfully injected with gene-specific CRISPR and an antisense morpholino targeting the *vwa1* protein-coding transcripts exon 1–2, which contained a blue-emitting fluorescent tag at the 3′ end (Supplementary Fig. 7A). Investigation of the effect of different concentrations of *vwa1* MO showed a dose-dependent phenotypic severity indicating the specificity of *vwa1* knockdown. The altered splicing had a negative effect on survival rate compared to controls. For both models, the examined groups’ gross morphology was comparable to the control group (Supplementary Fig. 7B).

**Figure 5:**
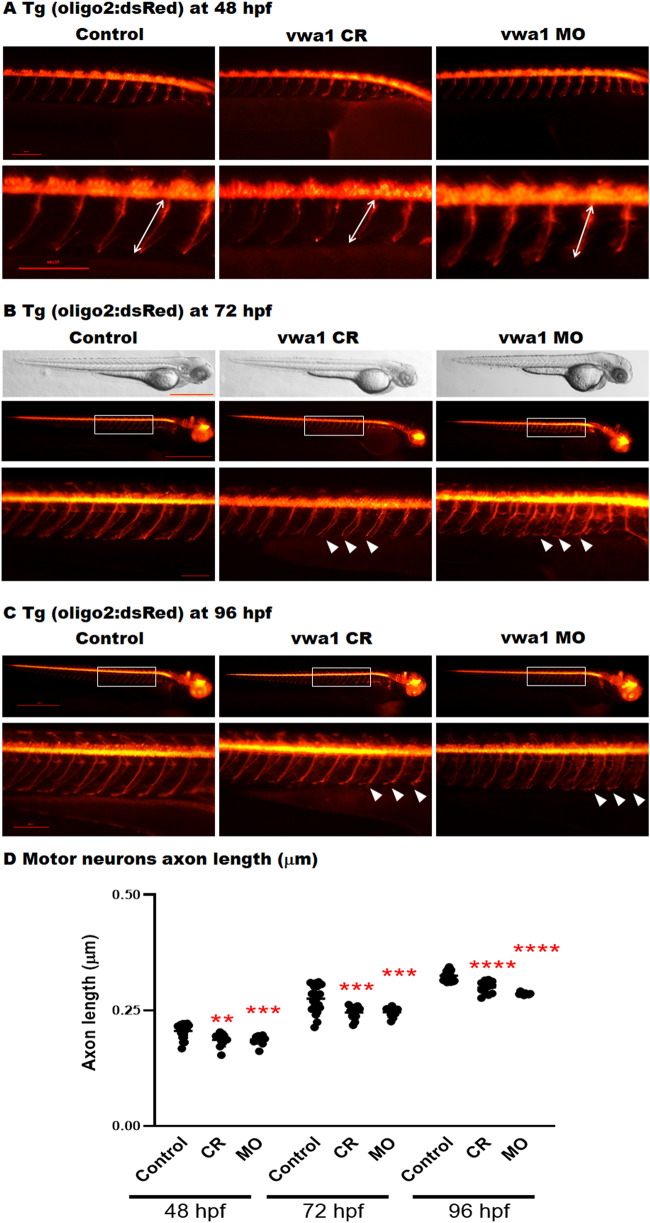
**The *vwa1* zebrafish model has impaired spinal motor neuron axon development.** Transgenic embryos [Tg(olig2:dsRed), expressing red fluorescent oligodendrocytes and motor neurons axons) were injected with *vwa1* CRISPR (knockout) or morpholino (MO, knockdown). Both vwa1 models displayed a significantly shorter motor axons in comparison to control axon growth. Representative embryos full-body lateral view and trunk lateral view for the examined groups at: (**A**) Spinal cord motor neurons examined at 48 hpf [enlarged images *below* showing the measurement of axon length from the base of the spinal cord to the end of the motor neuron (white arrow)]; (**B**) 72 hpf, examined spinal motor neurons (white box) and aberrant developing axons (white arrowheads); and (**C**) 96 hpf. (**D**) The *vwa1* model resulted in impaired motor neurons axon growth in comparison to control axon growth. Spinal motor neurons axon length was measured for 8–10 axons per embryo starting from axon number 5. Vwa1 Crispants (*n *=* *29) and morphants (*n *=* *26) showed significantly shortened axons compared to controls (*n *=* *22). Zebrafish imaging using Lumar 12 microscope (whole body images, scale bar = 40 mm and axon images, scale bar = 10 mm), *t*-test was used for statistical analysis. Spinal motor neuron images at a magnification of ×150, scale bar = 10 µm. Statistical analysis was performed with GraphPad Prism 8.0.

Phenotypic assessment of the *vwa1* crispants/morphants demonstrated a prominent effect on motor neurons including shorter axons and abnormal growth. Aberrant axon structures were observed from Day 3 and there was a significant reduction in spinal motor neuron growth measured through development at Days 2–4 (Fig. 5). It was observed that *vwa1* knockdown/knockout caused apparent developmental deformities in developing motor neurons (Supplementary Fig. 8). The examined primary motor neurons showed reduced axonal branching innervating the muscle fibres and aberrant secondary motor neurons with severe defects in the middle and caudal axons of  secondary motor neurons with some early truncation of these axons.

The skeletal muscle structure was evaluated in developing somites using phalloidin stain for filamentous actin (F-actin). Control groups displayed densely packed and organized muscle fibres, while the zebrafish *vwa1* model revealed sparser fibre arrangement, with the appearance of some disorganized myofibres. Moreover, examination of neuromuscular junctions, by targeting the acetylcholine receptors (AChR) using alpha-bungarotoxin stain, revealed significantly reduced synaptic formation in the skeletal muscles (Supplementary Fig. 9). These results established that *vwa1* is required for the proper organization of skeletal muscles and in the formation of neuromuscular junctions.

Additionally, the gross morphological appearance of *vwa1* crispants/morphants displayed a different cartilage patterning. Zebrafish *vwa1* also appears to be required for jaw joint development and helped pattern the ventral cartilage. The structure and area of the arches Meckel’s and palatoquadrate were irregular in the *vwa1* model (Supplementary Fig. 10A). The size of the jaw from the Meckel’s to the interhyal structure was also significantly reduced (Supplementary Fig. 10B).

Finally, locomotor behaviour was assessed in the zebrafish CRISPR *vwa1* knockout model. The model exhibited no impact at the first neuronal motor spontaneous movements at 1 day old (Supplementary Fig. 11). However, a consistent trend developed at 5 days old towards: (i) reduced total distance moved over time; (ii) reduced velocity; and (iii) increased frequency of non-movements from centre point when compared to control group (Supplementary Fig. 12A–C).

## Discussion

Over the past decade, availability of next-generation sequencing data for large patient cohorts has facilitated the discovery of numerous genes responsible for Mendelian disorders. It is likely that most of the ‘low-hanging fruit’ has been gathered already and in the future, discovery of novel monogenic disease entities will be limited to increasingly rare conditions, to syndromes where the causative variant is non-coding or where complex rearrangements that are difficult to detect with short-read technologies are involved. In this study, using data from the 100KGP, through international collaboration and by targeted sequencing, 17 individuals from 15 families harbouring biallelic variants in *VWA1* were identified.

The major disease-associated allele in *VWA1* involves an insertion of GGCGCGGAGC at the end of exon 1. According to the global allele frequency in gnomAD v3, the high frequency of this founder mutation (89/140 632) is comparable with other well-known variants seen in related autosomal-recessive conditions (Supplementary Table 7), coming just after the recently described p.A253Qfs*27 in *SORD* (623/142 588), p.I41T in *FIG4* (164/143 266) and p.R954* in *SH3TC2* (94/143 270). In European populations, the allele frequency of p.G25Rfs*74 rises to almost 1/1000. The 0.091% (58/63 526) allele frequency reported in gnomAD for non-Finnish Europeans is in line with the 0.087% (188/215 908) in the GeneDx exome database, the 0.094% (112/118 908) seen in the 100KGP project (Supplementary material and Supplementary Table 1) and the 0.102% (82/80 048, all heterozygous) in UK Biobank Exome sequencing data (https://pdgenetics.shinyapps.io/VariantBrowser), consistent with the variant being widespread in Europeans. Given this consistent allele frequency and the support from the WARP mouse model published over 10 years ago (Allen *et al.*, 2009), we were initially surprised that this disease gene had evaded discovery until now. The reasons for this seems to be primarily of a technical nature due to high GC content in *VWA1* (Supplementary material). Inefficient capture of this locus is observed in exome sequencing, the methodology most widely used for many clinical diagnostic laboratories, but this is less problematic for whole genome sequencing methods (Supplementary Fig. 13). The p.A253Qfs*27 in *SORD* was also previously overlooked likely due to technical reasons, although in that case it was likely due to a highly homologous pseudogene (Cortese *et al.*, 2020).

Patient enrolment to the 100KGP spanned >200 different recruitment categories (Turnbull *et al.*, 2018). Hereditary motor neuropathy was not present in this list of available options and the highest number of *VWA1*-positive cases was instead seen in the CMT group, for an incidence of 0.85% (6/708; Table 1). Revising the dominator to consider only a single affected individual per family and removing individuals of non-Caucasian ethnicity, the incidence rises to 1.12% (6/535). The lower frequency observed in the replication cohort (3/1341) may reflect differences in the ethnic background of the latter cohort (largely of German origin) and the degree to which prior genetic testing had excluded known neuropathy genes.

The uniform coverage afforded by the genome sequence data available for a subset of families allowed us to perform high-resolution analysis of haplotype sharing in individuals. These results confirmed p.G25Rfs*74 to be a founder mutation that may have arisen >7000 years ago. In tandem with the uniform coverage across *VWA1*, our study highlights the advantages of genome sequencing over exome sequencing approaches.

Historically, the identification of novel autosomal-recessive disease genes has often come from linkage-analysis and genetic co-segregation in large, often consanguineous families. In contrast, the majority of families in the present study are singletons and thus support for pathogenesis is required from different sources. Increasing availability of large standardized clinical genome datasets has meant that novel disease genes can be identified by looking for enrichment of severe biallelic alleles above expectation and for phenotypic similarity between patients harbouring putatively damaging variants in the same candidate gene. For instance, the Deciphering Developmental Disorders (DDD) study identified four novel recessive genes by performing formal statistical assessment across all genes for the likelihood of sampling the observed genotypes and combined this with detailed analysis of Human Phenotype Ontology (HPO) terms (Akawi *et al.*, 2015). Although the *VWA1* patients from the 100KGP were identified by a straightforward candidate gene analysis following sharing of preliminary results from Family 11, enrichment of homozygosity above expectation and the phenotypic similarity of patients retrospectively lends support to the p.G25Rfs*74 variant playing a role in pathogenesis. Given the estimated allele frequency in Europeans of <1/1000, the observation of the founder variant in seven homozygous individuals within the 100KGP would be unlikely under the null-hypothesis. Our unbiased analysis across the 100KGP was also significant in terms of the phenotypic similarity seen in the identified patients. The 100KGP recruited across several rare disease areas, of which peripheral neuropathies were just one component (Table 1). It is therefore notable that our systematic analysis did not detect any severe biallelic *VWA1* variants in other disease categories.

When a novel disease gene is implicated through a single founder mutation, there is always a concern that there may be an unobserved pathogenic variant on the same haplotype (MacArthur *et al.*, 2014). The coverage uniformity of genome sequencing means unobserved variants are less likely than would be the case through exome sequencing, but still does not 100% rule out that a detected variant has been overlooked. It is therefore reassuring that we identified three other variants predicted to result in premature termination codons. The p.M155Cfs*51 variant lies in the VWFA domain and is supported by co-segregation (Fig. 1A and B). In contrast, p.R293Sfs*58 and p.Q367* lie in the first and second fibronectin type III domains respectively and were identified in single affected individuals. For the latter two variants, even if the truncated protein is stable, 153/423 or 79/423 of the wild-type amino acid sequence would be missing once the 22-amino acid signal sequence is removed (Fig. 1B). Together with the immunoblotting results presented here (Fig. 4B), the absence of homozygous loss of function alleles in gnomAD and the results from the knockout mouse model, where the mutant allele comprised a targeted gene replacement (Allen *et al.*, 2008), these set of variants argues in favour of a loss of function disease mechanism. We note that functional studies would be required to confirm pathogenicity for p.S74R and p.Y364N alleles (Supplementary Table 1) and to support our structural modelling results which suggest both substitutions to be destabilizing.

Like many genomic resources, certain global ethnic categories are under-represented in data from 100KGP and gnomAD. We were therefore interested to note that in the internal CentoMD^®^ database (Trujillano *et al.*, 2017), the reciprocal 10 bp deletion (c.62_71del10) was present at an allele frequency of 71/136 000 (mostly in individuals of Middle Eastern ancestry) and so more common than c.62_71dup10 (26/136 000). Although no biallelic cases were identified, this shorter allele also predicts a premature stop codon (p.G21Afs*12) and so we speculate this may represent a second disease-causing founder mutation at the same locus.

The common clinical denominator in the presented series appears to be an early-onset slowly progressive non-length-dependent hereditary motor neuropathy. Albeit the distal presentation with foot deformities and distal weakness, the progression to the proximal muscle groups, which in some cases is more prominent than distal, raised in many instances the suspicion of non-length dependent neuropathy. Impaired nociception observed in WARP-null mice was not frequently seen in the WARP-deficient individuals from this series. A subset of paediatric cases in our cohort with only distal lower limb weakness, wasting, and foot contractures resembled the typical distal hereditary motor neuropathy type I presentation (Garg *et al.*, 2017). Although, with advancing age, weakness in the present series tended to progress proximally. There was some degree of overlap with spinal muscular atrophy with lower extremity predominance caused by variants in *DYNC1H* and *BICD2*. Specifically, the weakness that commenced and remained predominant in the lower limbs affecting the proximal more than distal muscles, character of the tendon contractures, foot and spinal deformities, and forward stood posture (Rossor *et al.*, 2015; Scoto *et al.*, 2015). However, in our series, the upper limbs were involved in more than half of the cases irrespective of the disease severity, unlike spinal muscular atrophy with lower extremity predominance.

Most of the EMG and over half of the muscle histology studies evidenced clear neurogenic changes. Hence, neurogenic muscle changes seem to be common in the VWA1 presentation, and it is likely for the observed myopathic process in a few cases to develop on the background of chronic muscle denervation. Of note, all of the histological features classically attributed to myopathy were abundantly found in the chronically denervated muscles from the poliomyelitis patients (Drachman *et al.*, 1967). It has been shown that myopathic changes may coexist with the classical pattern of denervation atrophy or dominated the biopsy picture. Similar to our study, several cases from *DYNC1H* and *BICD2* gene-positive cohorts revealed myopathic muscle biopsies (Rossor *et al.*, 2015; Scoto *et al.*, 2015).

WARP and collagen VI are co-expressed in the endomysium surrounding muscle fibres. However, WARP-null mice showed that WARP does not have a critical role in stabilizing the ECM localization of collagen VI in muscle as WARP-deficient mice expressed no histological or behavioural evidence of muscle pathology (Allen *et al.*, 2009). Consistent with this, immunostaining muscle biopsies of *VWA1* patients for collagen VI appeared normal in our study. Overall, the clinical and functional evidence from this study has shown that WARP deficiency in humans is likely to result in a non-length dependent motor axonal neuropathy. Characterization of further cases with WARP deficiency will improve our understanding of the phenotype range associated with this condition.

Comparative genome analysis has shown that >80% of human genes linked to human monogenic disease have an orthologue in zebrafish (Howe *et al.*, 2013). Together with the short generation time, high levels of fecundity, rapidly developing transparent embryos and ease of manipulation with MO technology, the zebrafish has become a powerful model organism for studying vertebrate developmental biology (Veldman and Lin, 2008). In this study we demonstrated that defective *vwa1* leads to subtle changes in motor axonal growth/density at very early stages of development (48–96 hpf) that correspond to the early stages of human neuronal development. Indeed, recent papers highlight the embryonic origin of many neuropathies and this may potentially explain the involvement of *VWA1* in the CNS malformations associated with 1p36 deletion syndrome (Shiba *et al.*, 2013). Zebrafish results were also consistent with the mouse model, with its impaired fine motor coordination (Allen *et al.*, 2009) and the relatively mild neuropathy seen in human patients described here. Overall, the defective myofibres’ organization, neuromuscular junctions, secondary motor neuron impairment and reduced larval locomotion may reflect the early vwa1 neuropathy. These observations encompass the specific degeneration of developing motor neurons leading to muscle weakness. The decrease in the area of jaw structures seen in zebrafish, although not a finding observed in patients, is notable given the high expression of WARP in chondrocytes (Fitzgerald *et al.*, 2002).

In summary, we describe a novel autosomal-recessive condition whereby biallelic *VWA1* variants lead to a hereditary non-length dependent motor neuropathy. Support for this genotype-phenotype correlation includes experiments using two different model organisms, an excess of homozygous founder alleles and by genetic co-segregation in one large Afghani kindred. Our detailed clinical characterization of the present patient cohort will facilitate targeted testing on suitable patient cohorts and extrapolation from 100KGP suggests this gene may explain up to 1% of unsolved cases of sporadic and hereditary neuropathy in European populations. Reassessment of unsolved exomes using lower QC filters at this locus may yield additional diagnoses.

## Supplementary Material

awaa420_Supplementary_Data

## Appendix 1

### The Genomics England Research Consortium

Full details are provided in the Supplementary material.

John C. Ambrose, Prabhu Arumugam, Emma L. Baple, Marta Bleda, Freya Boardman-Pretty, Jeanne M. Boissiere, Christopher R. Boustred, Helen Brittain, Mark J. Caulfield, Georgia C. Chan, Clare E. H. Craig, Louise C. Daugherty, Anna de Burca, Andrew Devereau, Greg Elgar, Rebecca E. Foulger, Tom Fowler, Pedro Furió-Tarí, Adam Giess, Joanne M. Hackett, Dina Halai, Angela Hamblin, Shirley Henderson, James E. Holman, Tim J. P. Hubbard, Kristina Ibáñez, Rob Jackson, Louise J. Jones, Dalia Kasperaviciute, Melis Kayikci, Athanasios Kousathanas, Lea Lahnstein, Kay Lawson, Sarah E. A. Leigh, Ivonne U. S. Leong, Javier F. Lopez, Fiona Maleady-Crowe, Joanne Mason, Ellen M. McDonagh, Loukas Moutsianas, Michael Mueller, Nirupa Murugaesu, Anna C. Need, Peter O’Donovan, Chris A. Odhams, Andrea Orioli, Christine Patch, Mariana Buongermino Pereira, Daniel Perez-Gil, Dimitris Polychronopoulos, John Pullinger, Tahrima Rahim, Augusto Rendon, Pablo Riesgo-Ferreiro, Tim Rogers, Mina Ryten, Kevin Savage, Kushmita Sawant, Richard H. Scott, Afshan Siddiq, Alexander Sieghart, Damian Smedley, Katherine R. Smith, Samuel C. Smith, Alona Sosinsky, William Spooner, Helen E. Stevens, Alexander Stuckey, Razvan Sultana, Mélanie Tanguy, Ellen R. A. Thomas, Simon R. Thompson, Carolyn Tregidgo, Arianna Tucci, Emma Walsh, Sarah A. Watters, Matthew J. Welland, Eleanor Williams, Katarzyna Witkowska, Suzanne M. Wood, Magdalena Zarowiecki.

### Supplementary material

Supplementary material is available at *Brain* online.

## Abbreviations

100KGP: 100K Genomes Project
ECM: extracellular matrix
