# Supplementary material for: An ancestral 10-bp repeat expansion in VWA1 causes recessive hereditary motor neuropathy
Source: Brain. 2021 Jan 18;144(2):584–600. doi: 10.1093/brain/awaa420 (PMC8263055; doi:10.1093/brain/awaa420)
Supplement: awaa420_Supplementary_Data [file awaa420_supplementary_data.zip › brain-2020-01257-File008.pdf]

Family ID	Family 1	Family 2	Family 3	Family 4	Family 5
**Individual**	Patient 1	Patient 2	Patient 3	Patient 4	Patient 5
**DNA Variant**					
***VWA1* variant at cDNA level (NM_022834.5)**	c.62_71dup10	c.62_71dup10	c.[62_71dup10];[222T>G]	c.62_71dup10	c.62_71dup10
***VWA1* variant at protein level**	p.(G25Rfs*74)	p.(G25Rfs*74)	p.[G25Rfs*74];[S74R]	p.(G25Rfs*74)	p.(G25Rfs*74)
**Epidemiological data**					
**Ethnic Group**	British	N/A	White British	British (Maternal: White; Paternal: Afro-Carribbean)	White British
**Gender**	F	F	M	M	M
**Consanguinity**	No	No	No	No	No
**Family history**	No	No	No	No	No
**Current age (years)**	50	68	70	8	50
**Age at onset**	18 months	14 months	2 years	4 years	2 years
**First presentation**	Tiptoe walking	N/A	Abnormal curled toes as a toddler	Abnormal foot shape and progressive gait difficulties	Childhood, toe-walking as a child
**Age of walking**	18 months	18 months	Normal	10 months	N/A
**The age when UL became involved**	40 years	N/A	N/A	NA	N/A
**Myalgia**	Yes	No	No	No	Yes
**Frequent falls**	Yes	No	No	No	No
**Loss of ambulation (age)**	No	No	No	NA	No
**Age at last examination (years)**	50	65	70	6.5	48
**Scoliosis**	No	No	No	No	No
**Lumbar hyperlordosis**	No	No	No	No	No
Pes cavus	Yes	Yes	Yes	Yes	NA
**Talipes equinovarus**	No	No	No	No	NA
**Tendon contractures**	Yes Achilles tendon	No	No	Yes, Achilles tendon	Yes
**Scapular winging**	No	No	No	No	No
**Cranial nerves**	Anisocoria	Normal	Anisocoria	Normal	Normal
**Tongue fasciculations**	Yes, mild	NA	Tongue wasting	No	No
**Pattern of muscle weakness**					
**UL proximal**	Mild	Mild	Moderate	Normal	Mild

**UL distal**	Mild	Normal	Mild	Normal	Normal
**LL proximal**	Moderate to Severe	Moderate	Moderate	Normal	Mild
**LL distal**	Mild	Moderate	Mild	Contractures	Normal
**Muscle wasting (part of the body)**	Yes, hands and thighs	No	Thighs and below knee	Yes, distally LL	No
**Foot drop**	Yes	No	No	Yes	N/A
**Myopathic gait**	Yes	Yes	No	No	Yes
**Muscular hypotonia**	Yes	No	No	No	No
**Hyporeflexia**	Yes	No	Yes	No	No
**Hyperreflexia at the knees**	No	No	No	No	No
**NCS**	MAN	MAN	MAN	MAN	MAN
**EMG**	CNC	MC	N/A	CNC	MC
**Muscle biopsy changes**	N/A	Myopathic	N/A	Neurogenic	Myopathic
**Muscle MRI**	Fatty replacement	Compatible with myopathy	N/A	N/A	N/A

Supplementary Table 6: Detailed clinical information

F= female; M= male; N/A= not available; NA= not applicable; UL= upper limbs; LL= lower limbs; MAN= Motor axonal neuropathy; CNC=
resonance imaging

Family 6	Family 7	Family 8	Family 9	Family 10	Family 11	Family
Patient 6	Patient 7	Patient 8	Patient 9	Patient 10	Patient 11	Patient 12
c.62_71dup10<br>p.(G25Rfs*74)	c.62_71dup10<br>p.(G25Rfs*74)	c.[62_71dup10];[1099C>T]<br>p.[G25Rfs*74];[Q367*]	c.62_71dup10<br>p.(G25Rfs*74)	c.[62_71dup10];[1090T>A]<br>p.[G25Rfs*74];[Y364N]	c.62_71dup10<br>p.G25Rfs*74	c.62_71dup10<br>p.(G25Rfs*74)
White Caucasian	White British	White British	White British	Italian/Scottish/English	Italian, Irish, Dutch, Scottish, German	Caucasian
M	F	F	M	F	M	M
No	No	No	No	No	No	No
Mother walking impairment from 42 years	Maternal grandmother with suspected CMT	No	No	No	No	Affected identical twin
40	10	70	10	6	8	57
4 years	3 years	2 years	2 years	2 years	Birth	congenital
Falls and left foot turned in	Asymmetric foot drop	N/A	Not walking. Arthrogryposis	N/A	Equinovarus and bilateral tight heel cords	Congenital club feet
2.5 years	Normal	Normal	2.5 years (with aid of calipers and Piedro boots)	17 months	15 months	N/A
Childhood	8 years	NA	NA	NA	NA	N/A
No	No	Yes	Yes	Yes	No	Yes
Yes	N/A	No	Yes	No	Yes	NA
Yes	NA	No	No	No	No	Yes
33	10	67	10	6	6	56
No	Yes	No	No	No	No	N/A
No	Yes	Yes	N/A	No	Yes	Yes
Yes	Yes	Yes	No	Yes	No	N/A
Yes	No	No	Yes	Yes	Yes	Yes
Yes, possible shoulder contractures	N/A	No	N/A	No	Yes, Bilateral heel cord, hamstrings, hip flexors	Yes, Elbows and wrists
No	No	No	No	No	Yes	N/A
Normal	Normal	Normal	Normal	Normal	Normal	Partial ophtalmoparesis, dysarthria
No	No	No	No	No	No	No
Normal	Moderate	Mild	Normal	Normal	Mild	Severe

Normal	Mild	Normal	Normal	Normal	N/A	Moderate
Mild	Moderate	Mild	Mild	Normal	Mild	Severe
Contractures	Moderate	Mild	Severe	Moderate	Moderate	Moderate
Yes, lower legs to mid-calf	No	Yes, distal LL	Yes, LL	Yes, dystal LL	Yes, LL	N/A
NA	Yes	No	Yes	Yes	Yes	N/A
No	No	No	N/A	N/A	Yes	N/A
No	NA	No	N/A	No	No	N/A
Yes	Yes	Yes	N/A	No	Yes	N/A
No	No	No	No	Yes	No	No
MMSN	MAN	MAN	MAN	MAN	MAN	MAN
ADC	N/A	CNC	N/A	CNC	CNC	CNC
Neurogenic	N/A	N/A	Neurogenic	N/A	N/A	Myopathic
N/A	N/A	N/A	N/A	N/A	Increased signal intensity on T1	N/A

= chronic neurogenic changes; ACD= acute denervation changes; MMSN= multifocal motor and sensory neuropathy; MC= myopathic [illegible]

12	Family 13		Family 14	Family 15
Patient 13	Patient 14	Patient 15 (Uncle)	Patient 16	Patient 17
c.62_71dup10	c.456delC	c.456delC	c.62_71dup10	c.[62_71dup10];[879delG]
p.(G25Rfs*74)	p.M155fs*50	p.M155fs*50	p.(G25Rfs*74)	p.[G25Rfs*74];[R293Sfs*58]
Caucasian	Afghani	Afghani	N/A	German
M	M	M	M	F
No	Yes	Yes	N/A	No
Affected identical twin	Yes (affected uncle)	Yes	N/A	No
57	11	42	83	24
congenital	2 years	5 years	N/A	1 year
Congenital club feet	Hip dislocation and developmental delay and recurrent falling	Recurrent knee dislocation	N/A	Gait abnormality, frequent falls, leg asymmetry due to the inner rotation of left hip
N/A	19 months	NA	N/A	Started walking at 12 months, fully able to walk at 4-5 years, balance control at 6-7 years
N/A	Recent	Recent	N/A	14-15 years
Yes	No	No	N/A	No
NA	Yes	No	N/A	Yes (previously, not anymore)
Yes	No	No	N/A	No
56	11	42	N/A	24
N/A	Yes	Yes	N/A	Yes (mild, thoracal spine)
Yes	Yes	Yes	N/A	No
N/A	Yes	Yes	Yes	No
Yes	No	No	N/A	No
Yes, Elbows and wrists	Yes, Bilateral Achille, knee and hip contracture	Yes, Bilateral Achille, knee, fingers and hip contracture	N/A	No
N/A	Yes	No	N/A	No
Dysphagia	Normal	Normal	N/A	Normal
No	No	No	N/A	No
Severe	Mild	Normal	N/A	Normal

Moderate	Mild	Mild	N/A	Mild
Severe	Mild	Normal	N/A	Mild
Moderate	Severe	Severe	N/A	Severe
N/A	Yes, LL	Yes, LL	N/A	No
N/A	Yes	Yes	N/A	Yes
N/A	Yes	Yes	N/A	No
N/A	No	No	N/A	No
N/A	No	Yes	N/A	Yes
No	Yes	No	N/A	N/A
MAN	MAN	N/A	N/A	MAN (mild)
CNC	MC and neurogenic changes	N/A	N/A	CNC
N/A	N/A	N/A	N/A	Neurogenic
N/A	Fatty replacement	N/A	N/A	N/A

changes; NCS= nerve conduction study; EMG=electromyography; MRI=magnetic
