# Supplementary material for: An ancestral 10-bp repeat expansion in VWA1 causes recessive hereditary motor neuropathy
Source: Brain. 2021 Jan 18;144(2):584–600. doi: 10.1093/brain/awaa420 (PMC8263055; doi:10.1093/brain/awaa420)
Supplement: awaa420_Supplementary_Data [file awaa420_supplementary_data.zip › brain-2020-01257-File009.pdf]

An ancestral 10bp repeat expansion in *VWA1* causes recessive hereditary motor neuropathy: Supplementary Case Reports

Family 1

The affected individual from Family 1 is a 50-year-old female, who was born at full term to the non-consanguineous White British parents. Her neonatal measurements were unremarkable. She acquired independent walking by 18 months of age when she was noticed to predominantly walk on tiptoes. Later she had developed progressive weakness in her hips that made her difficult to climb up the stairs and raise from the floor from childhood. She is currently unable to ascend from the squat and experiences frequent falls. Muscular weakness had gradually progressed to her upper limbs that had become noticeable from the age of 40 years. The disease has been having slow rates of progression allowing her to maintain independent ambulation up to current age and work as a barista at a coffee shop in London. Her past medical history was unremarkable except for chronic obstructive pulmonary disease leading to occasional episodes of shortening of breath. The cranial nerves examination revealed anisocoria, unreactive left pupil, and the sluggish right pupillary response to the light. The neuromuscular examination was suggestive of non-length dependent hereditary motor neuropathy (Supplementary video). More specifically, there were mild tongue fasciculations, limb hypotonia, and mild amyotrophy on the bottom of the thighs and in the hands. Muscle power was significantly reduced in hip flexors bilaterally (grade 2) and mild weakness (grades 4 and 4+) was present in hip extension, knee flexion, foot dorsiflexion, and foot inversion. Mildly reduced power was also found in the upper limbs (grades 4 and 4+). While her ankle jerks were bilaterally absent, the tendon reflexes from knees were elicited only with reinforcement. Upper limb tendon reflexes were depressed on the left side. There was mild distal hand tremor with arms outstretched. She had *pes cavus*, mild foot drop, Achilles tendon contractures with down going plantars, and inability to walk on heels. There was paresthesia with the normal sensory examination. She had forward stood posture and no signs of ataxia. EMG showed evidence of widespread chronic denervation changes. Nerve conduction studies showed signs of motor axonal neuropathy. Magnetic resonance imaging of the upper and lower limbs revealed mild-to-moderate bilateral and symmetric fatty replacement uniformly involving all the proximal and distal muscles of the lower limbs. A mild and selective fatty replacement was also found in the upper limbs involving bilaterally deltoid, the distal component of the biceps brachii, and triceps muscles.

Family 2

A 68-year-old female was reported to have a delay in unassisted gait acquisition due to slowly progressive muscle weakness in the lower limbs. She was noticed to have weak legs from the age of 14 months that had delayed the start of independent walking until the age of 18 months. Her physical examination at the age of 65 years was remarkable for hypertension. Upon neuro-muscular examination, she was found to have symmetric muscle weakness predominantly affecting the proximal muscles of the lower and upper limbs (grade 3 on neck flexion, hip extension, and knee flexion; grade 4 on shoulder abduction and hip flexion; grade 4+ on knee extension; grade 4- on elbow extension). Her feet appeared also weak on dorsiflexion (grade 4 on the left foot and grade 3 on the right foot), foot plantarflexion (grade 4 bilaterally), and eversion (grades 4 on the left foot and grade 3 on the right foot). Despite her *pes cavus* and the distal muscle weakness in the lower limbs, she was able to walk on toes/heels and had not experienced frequent falls. Strengths of the hand muscles, tendon reflexes, muscle tone, and sensation

remained intact. She could walk independently with proximally weak gait. Electromyography was reported as compatible with myopathic changes but the formal report was not available for review. Muscle MRI was interpreted as suggestive of Bethlem, although the images were not available for our review. Muscle histology showed marked fibre hypertrophy and increased fibrosis with endo and perimysial fatty infiltration. Labelling for collagen VI appeared normal. Prior genetic testing included analysis of genes linked to myofibrillar myopathy, facioscapulohumeral muscular dystrophy, *TTN*, *COL6A1/2/3*. Except for a heterozygous c.3754C>T (p.Arg1252Cys) variant in *COL6A3* (NM_004369.3) which is of uncertain significance, no likely causative variants were identified.

Family 3

The index case is a 70-year-old White-British male, a product of uneventful pregnancy and delivery, who was noticed to have abnormally curled toes as a toddler. He did not have a motor delay, however later from adolescence he had noticed slowly progressing fatigue and weakness going up the stairs. From the age of 50 years weakness in the lower limbs had started rapidly worsening leading to occasional falls with the frequency of ones per month. His physical examination was revealing for hypertension and the sensation of dyspnea. Cranial nerves examination showed the presence of anisocoria, mild squint, and tongue wasting. Neuromuscular work-up found globally and symmetrically reduced muscle power in the upper and lower limbs that was more pronounced in hip flexion and elbow extension (grade 3). His lower limb muscles were amyotrophic in the quadriceps and below the knees. Muscle tone and sensation were normal but tendon reflexes were uniformly absent and Babinski sign was negative. He had bilateral *pes cavus* and despite the long-standing disease course remained ambulatory with the help of walkers on the wheels. Forward stood posture was noticed. Electrophysiological studies showed signs of a chronic motor axonal neuropathy consistent. *SMN1* gene testing was negative.

Family 4

An affected individual from Family 4 is an 8-year-old boy born at full term to a White-British female and Afro-Caribbean male. He had unremarkable postnatal development and acquired motor milestones at a normal age. However, from the age of 4 years he had started experiencing progressive gait difficulties due to distal weakness in the lower limbs. Around the same time, his parents noticed the manifest of rapidly progressing feet deformities. Since then, pain and blisters in his feet secondary to foot deformities had been the main problems. He has developed bilateral *pes cavus* and Achilles tendon contractures but no spinal deformities were found upon physical examination. Neurological examination revealed an intact cranial nerve function, muscle tone, and sensation. Power was reduced only distally in the lower limbs leading to mild foot drop, distal muscle wasting, and absent ankle jerks. Achilles tendon contractures limited the assessment of the feet in the frontal plane and the patient had marked toe walking with an inability to heel walking. His Creatinine phosphokinase ranged between 775 IU/l - 1628 IU/l. Electrophysiology was abnormal with the signs of motor axonal neuropathy, bilateral neurogenic changes and preserved sensory conduction velocities. Muscle biopsy showed clear neurogenic changes with a dystrophic component. Ultrasound of the thigh muscles demonstrated normal echogenicity except for the possibility of a mild increase within the vastus intermedius. His brain and spine MRI were unrevealing.

Family 5

A 50-year-old White-British male with no known family history of neuromuscular disorders was reported to have episodes of toe walking during childhood that had spontaneously resolved. His later motor performance over adolescence and adulthood until the mid-forties was unremarkable. By the age of 44 years he has started experiencing slowly progressive myalgia and weakness in the limb-girdle muscles. His physical examination at the age of 48 years was unremarkable and the neuromuscular examination showed proximal limb muscle weakness (left shoulder abduction grade 3, left hip flexion/extension grade 5, knee extension grade 4 bilaterally, knee flexion grade 4+ bilaterally). Muscle tone, tendon reflexes, and sensation appeared normal with no foot deformities and extensor plantar response. His gait was myopathic and independent ambulation was preserved with a need for a wheelchair only for long distances. Creatine kinase levels were increased up to 1000 U/L. Electromyography and muscle biopsy were reported to suggest myopathic changes, albeit the formal report for EMG was not available for review. Due to the late-onset limb-girdle muscle weakness and consistent myopathic changes on electrophysiology and muscle biopsy the clinical diagnosis of probable limb-girdle muscular dystrophy was made. WES revealed variants of unknown significance in GSD panel, *ANO5, CAPN3, GNE* and *VCP* genes.

Family 6

An affected individual from Family 6 is a 40 year-old Caucasian man with a history of delayed walking (2.5 years old), which had later progressed to the equinovarus foot deformity and falls starting from the age of 4 years. Around the same age, he had developed slowly progressive muscle weakness in the distal lower limb muscles. The foot deformity was corrected by surgery and he had gradually lost independent ambulation by the age of 30 years. His mother was reported to develop walking problems from 42 years old. She was seen by a neurologist and nerve conduction studies in 1991 showed some slowing in sensory conduction only but no formal diagnosis was made. Upon neurological examination at the age of 33 years, the patient was found to have mild proximal weakness in the lower limbs and little movements in feet due to severe equnovarus-related corrective foot surgery. *Pes cavus* and distal muscle wasting in the lower limbs were also present. Muscle tone in the lower limbs was increased but knee jerks were absent. Muscle power in the upper limbs remained intact, although there were signs suggestive of muscle hypertonia and shoulder contractures with absent supinator reflexes. Sensory examination revealed intermittent paresthesia, reduced pinprick sense up to the wrists and the mid part of the shins, as well as impaired vibration sense up to the shoulders and the knees. Nerve conduction studies (NCS) in 1980 was compatible with axonal motor neuropathy with preserved sensory conduction. Follow-up NCS in 2014 revealed largely asymmetric multifocal motor and sensory neuropathy restricted to the legs. This was partially attributed to a history of a car accident-related lower spinal cord injury. Electromyography conducted in 1980s showed fibrillation potentials, occasional fasciculations, and pseudo-myotonic units in the tibialis anterior muscles. Muscle biopsy in 1988 showed the signs of denervation. The genetic work-up by Bristol neuropathy panel was unrevealing.

Family 7

The index case is a 10-year-old girl born to the non-consanguineous White-British parents. There was not a history of motor delay, however from the age of 3 years she had started developing slowly progressive distal muscle weakness in the legs with the asymmetric right predominant foot drop. Muscle weakness had gradually ascended involving the upper limbs by the age of 8 years. Her maternal grandmother was reported to have possible Charcot-Marie-Tooth disease. On examination mild thoracolumbar scoliosis, lumbar hyperlordosis, and *pes cavus* were evident. She had mild proximal (grade 4) and moderate distal weakness (grade 3) in the lower limbs with bilateral foot drop. Mild symmetric weakness in the upper limbs was also revealing (elbow extension grade 4+) with rather weak neck flexors (grade 3+). Sensory examination was normal but tendon reflexes were universally suppressed. Nerve conduction studies (NCS) at the age of 3 years old showed small peroneal motor responses but this was insufficient for the diagnosis of neuropathy. Follow-up NCS at 5 years old revealed normal sensory studies with the asymmetrical common peroneal motor axonal loss. The Bristol IPN panel found a paternally inherited VUS in *MFN2* (NM_001127660.1); c.1452G>A.

Family 8

A 70-year-old White British female reports normal early motor development but from early childhood she had developed a progressive varus foot deformity. By the age of 7 years the foot deformity was corrected by surgery; however, from this time she recalls experiencing chronic pain in the legs with a particularly nocturnal troublesome throbbing sensation in her calves. Myalgia symptoms had settled by her thirties and she had been relatively stable until her mid-sixties. Hence, from this age she had started experiencing unsteadiness on her feet and felt that she had lost control over her ankles. She needed to hold onto things, particularly when going up the stairs due to the weakness in the feet and hips. She had also started going over on her ankles again. Her physical examination at the age of 67 years revealed arterial hypertension and lumbar hyperlordosis. There was distal limb muscle wasting with *pes cavus* and no foot drop. She had mild distal and proximal weakness in the lower limbs (grade 4 bilaterally). The upper limbs were noted to have only mild weakness on bilateral shoulder abduction. Tendon reflexes and sensation modalities were preserved with down going plantar reflex. Her gait was normal and based on her symptoms, she was thought to have distal hereditary motor neuropathy. Neurophysiology studies revealed sings of a mild motor axonal neuropathy with chronic neurogenic change in all the lower limb muscles examined. Normal sensory studies and no active denervation in any muscles examined.

Family 9

The affected individual is a 10-year-old male born at full term after unremarkable pregnancy to non-consanguineous White-British parents. Upon birth he was found to have mild congenital flexion contractures. After uneventful early post-natal development, by the age of 2 years he was noticed to have delayed walking and equinovarus foot deformities. He developed slowly progressive distal muscle weakness in the lower limbs and started walking by the age of 2.5 years with the aid of calipers and Piedro boots. Occasionally, he experiences pins and needles and pain in legs. He is known to have autistic spectrum disorders and difficulties in mathematics and literacy that lags him 3 years behind the normal academic performance. His physical examination at 10 years old did not reveal spinal deformities but bilateral talipes equinovarus was present for which he was awaiting surgery. Proximal and distal muscle weakness in the lower limbs with distal amyotrophy, foot drop, and normal

sensation were revealing upon his neurological examination. Muscle power remained intact in the upper limbs and his gait was wide-based and wobbly. Nerve conduction studies suggested an axonal motor neuropathy. Electromyography was unavailable. Muscle biopsy was consistent with neurogenic change with no abnormality of muscle fibers. He had normal creatine kinase, acylcarnitines, lactate, and Pompe's biochemistry. No evidence of mitochondrial respiratory chain defect was found. Head and spine MRI at age 6 years was normal. His condition was classified as paediatric motor neuronopathies. The Birmingham (WMRGL) neuropathy panel revealed a maternally inherited VUS in *MFN2* (NM_0178774.3) c.1403G>A, p.(Arg468His).

Family 10

The proband is a 6-year-old female born at full term to non-consanguineous parents of mixed Italian, English and Scottish origin. Concerns started with delayed walking and distal muscle weakness in the lower limbs. She started independent walking by the age of 17 months with casting. Her muscle weakness has been slowly progressing with precipitated myalgia but she is currently able to walk for long distances with orthotics and manage stairs with only occasional falls. Her current clinical examination reveals no spinal curvatures, normal cognition, and intact muscle power in the upper limbs. Muscle weakness in the lower limbs was present only distally with slight foot drop, *pes cavus*, and mild amyotrophy on the left leg. Sensory examination was normal and tendon reflexes were increased at the knees and ankles with negative Babinski. She had forward stood posture and symmetric gait but wide-based when running. Creatinine phosphokinase levels were normal and neurophysiology studies showed pure motor axonal neuropathy with neurogenic changes. Brain and spine MRI were unrevealing. The initial pursuit of the molecular diagnosis was negative for SMA, PMP22 duplication, and Frataxin gene expansion. NGS panel testing has found maternally inherited variant of unknown significance in *AARS*, c.461A>G; p.(Gln154Arg).

Family 11

The index case is an 8-year-old boy born at full term after unremarkable pregnancy and delivery to non-consanguineous parents of mixed European origin. He was born with bilateral equinovarus and tight heel cords that were later surgically corrected. Independent gait was acquired by 15 months of age (delayed due to foot deformity and toe walking). Later, he developed bilateral hip flexion contractures and tight hamstrings combined with slowly progressive hip girdle muscle weakness and distal muscle atrophy in the lower extremities. Currently he experiences frequent falls and needs to crawl when managing the stairs. His medical examination at 6 years old was revealing for waddling gait, bilateral femoral anteversion, and lumbar hyperlordosis. Distal more than proximal muscle weakness was identified in the lower limb examination (hip extension grade 4+, distal leg muscles grades 3- and 3+). Normal sensory examination and besides reduced ankle jerks, normal tendon reflexes were found. Electrophysiology studies showed evidence of moderate chronic length-dependent motor axonal neuropathy. Muscle ultrasound was abnormal in the lower extremities consistent with a neurogenic process. Muscle magnetic resonance imaging (MRI) of the lower extremities showed increased signal intensity on T1-weighted images whilst brain MRI was normal. Creatine phosphokinase ranged between 221-488 U/L. Chromosome SNP array and blood based mitochondrial genome were unrevealing.

Family 12

The index case is a 57-year-old Caucasian male with a history of motor delay, congenital club feet, and slowly progressive limb-girdle muscle weakness. He had later developed myalgia, elbow, and wrist contractures. His foot deformity was corrected by surgery. There was a relatively rapid deterioration of muscle weakness starting from the 5th decade of life. Up to the age of 50 years he used a wheelchair only for longer distances, from 53 years old he needed to use walkers for short distances, and the year after he had become wheelchair-bound. Dysarthria, hypertension, and obstructive sleep apnea have also precipitated from around the same age. His clinical examination revealed lumbar hyperlordosis, right eye exotropia, the limited elevation of both eyes, and limitation of left eye abduction. Muscular weakness was symmetrically pronounced on shoulder abduction and hip flexion (grade 2). Moderate weakness was present on knee and finger extension bilaterally (grade 3). Mild weakness was identified on neck flexion, elbow flexion, and wrist extension. He had an affected identical twin with exactly similar phenotypes except for swallowing difficulties. Nerve conduction studies showed signs of severe motor axonal neuropathy, low motor responses in tibialis posterior, normal sensory responses, and normal motor responses in the ulnar and median nerves. Needle electromyography showed old and chronic neurogenic changes with no evidence for denervation or spontaneous activity. Muscle biopsy demonstrated myopathic changes with marked fibre lobulation and variable degree of whorling of myofibrils combined with endomysial fatty infiltration. There were large areas of fibre grouping in this biopsy as well indicated a neurogenic aetiology. Creatine kinase ranged around 369 U/L. The index case and his affected twin were believed to have a combined limb girdle muscular dystrophy and axonal polyneuropathy.

Family 13

The index case is an 11-year-old boy born to a consanguineous Afghani family. He had a history of motor developmental delay and bilateral hip dislocation. From the age of 2 years he was noticed to have hip flexion contractures and experience frequent falls. From around the same age, he had started developing slowly progressive distal muscle weakness in the lower limbs gradually spreading proximally but leaving the distal muscles rather weaker. By the age of 10 years the muscle weakness had progressed to the upper limbs. Clinical examination at 11 years old revealed mild scoliosis, lumbar hyperlordosis, and bilateral contractures in all major joints of the lower limbs. Upon neurological assessment, cognition and cranial nerves appeared normal. *Pes cavus,* bilateral distal lower limb amyotrophy, foot drop, forward stood posture, and scapular winging were noticeable. Mild bilateral proximal and distal weakness was revealed in the upper limbs (grade 4) and power in the proximal lower limb muscles was mildly reduced to grade 4. Foot and toe extension bilaterally failed due to complete paralysis, while mild weakness was present on foot inversion and eversion. Besides absent ankle jerks, other tendon reflexes were normal with intact muscle tone and down going plantars. Distal pinprick and light touch sensory loss was present in lower limbs. He could walk unaided with bilateral foot drop. Nerve conduction studies revealed absent and reduced compound motor action potentials in the left and right peroneal nerves respectively. Electromyography showed a mixed myopathic and neurogenic pattern with profound myotonic discharges in the tibialis anterior muscles. Muscle magnetic resonance imaging was revealing for the fatty degeneration in all the calf muscles.

The index case had a similarly affected 42-year old paternal uncle presenting with childhood-onset slowly progressive distal muscle weakness and multiple contractures. The disease manifested in the uncle with

recurrent knee dislocations from the age of 5 years ensuing in the equinovarus deformity at the knees. The muscular weakness progressed to his distal upper limbs only by the fourth decade of life. Besides major joint contractures in the lower limbs he had also developed the finger contractures. His clinical examination was similar to his nephew's except for the preserved muscle power in the proximal limb muscles, no scapular winging, and absent tendon reflexes from lower limbs. In addition, sensory loss in all modalities was found in the lower limbs upon sensory examination.

Family 14

Limited clinical information is available for Family 14. The index case is an 83-year-old male with long-standing skeletal abnormalities and *pes cavus*. Increasing gait abnormalities have been observed over a 12 year period. Clinical examination signs were consistent with motor neuropathy whilst nerve conduction surveys show neurogenic changes. Spinal muscular atrophy was proposed as a possible diagnosis.

Family 15

The affected individual is a 24-year-old female born at full term to non-consanguineous German parents. Her neonatal measurements were in normal ranges. She had no delay gaining head control, sitting or crawling. She started to step at the age of 12-14 months, however independent walking was fully possible at the age of 4-5 years. The parents noticed a peculiar gait with frequent falls. They also noticed that her right foot was shorter than her left foot. The first detailed neurological assessment at 3 years of age including EMG/NCS and muscle/nerve biopsy revealed a distal axonal motor neuropathy. 5q-SMA was ruled out via genetic testing. Over the years, the patient received regular physiotherapy. Nonetheless, she developed a slight scoliosis, weakness in upper limbs (at 14-15 years of age), and her steppage gait caused by foot drop remained unchanged until today. No sensory deficits were mentioned in history. At the age of 19, her lower left leg was amputated following a traffic accident. She did not report any neurological deficits or deterioration of motor function after the accident.

On recent examination at 24 years of age, she described no peculiar neuromuscular restrictions except for the foot drop of the right foot and occasional back pain due to scoliosis. She did not complain of weakness in arms and hands, nor of problems with speech or swallowing. She does horse riding regularly and is able to walk 15 km a day without any complaints.

During examination, no cranial nerve deficits, tongue atrophy or fasciculation, bulbar signs or scapula alata were detected. She had almost full strength in arm flexion, but weakness of hand muscles (MRC 4-), in particular finger extension and abduction. There was also a mild weakness in hip flexion (MRC 4-) with normal hip extension. Particularly, she was not able to lift her right foot and toes (MRC 0). She could not walk on heels, but could perform toe walking. No muscle atrophy or foot deformity was obvious. Interestingly, we also observed several keloid formations on the wounds that remained after the muscle biopsy and traffic accident.

EMG showed chronic neurogenic changes, but no fibrillation potentials and/or positive sharp waves. Interference pattern was poor in the tibialis anterior muscle, whereas interference pattern of rectus femoris muscle and arm muscles were good. NCS were normal besides a compound motor action potential (CMAP) in lower normal range and reduced persistence of F-waves (2/10) of the right peroneal nerve. Creatine kinase level was at 535 U/l (N<170 U/l). Muscle biopsy performed at age 3 years revealed chronic-neurogenic tissue pattern with group atrophies and fiber type grouping. Nerve biopsy at the same age showed minor neuropathic changes with single hypomyelinating

fibres as a sign of cord damage and rarely detectable recently decaying axons as a sign of axonal damage. Her muscle ultrasound was normal.

In the family, the father has an early adulthood-onset arthrosis in various joints, including knees, mandibular, hips and hands. He has no neuromuscular symptoms. He is a heterozygous carrier of the 10bp insertion. There is a conspicuous predisposition to migraine on the maternal side, including the patient herself, her sister, her mother, her aunt, and her grandmother. The mother and sister are heterozygous carriers of the c.879del variant.
