# Supplementary material for: An ancestral 10-bp repeat expansion in VWA1 causes recessive hereditary motor neuropathy
Source: Brain. 2021 Jan 18;144(2):584–600. doi: 10.1093/brain/awaa420 (PMC8263055; doi:10.1093/brain/awaa420)
Supplement: awaa420_Supplementary_Data [file awaa420_supplementary_data.zip › brain-2020-01257-File011.pdf]

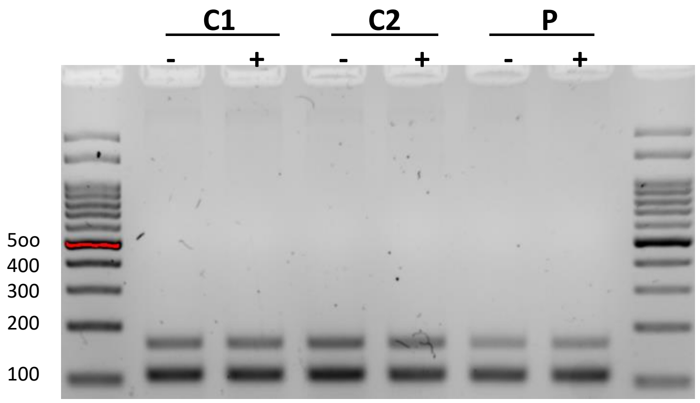
C1
C2
P
-
+
-
+
-
+
5oo
400
300
200
100

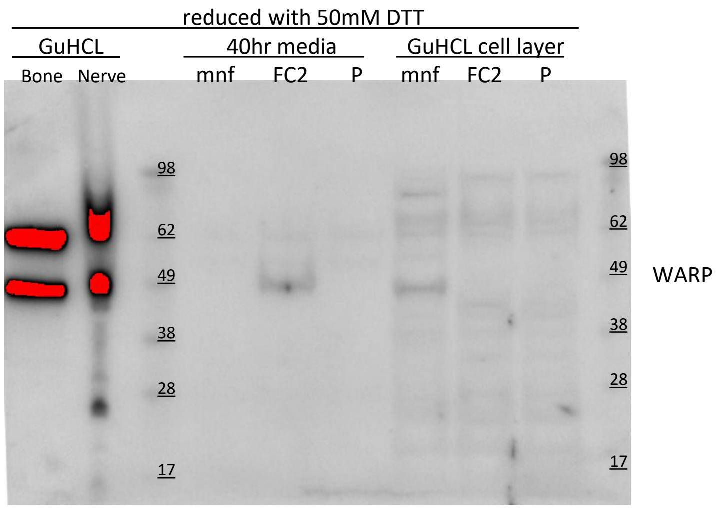
reduced with 50mM DTT
GuHCL
Bone
Nerve
40hr media
mnf
FC2
P
GuHCL cell layer
mnf
FC2
P
98
62
49
38
28
17
WARP

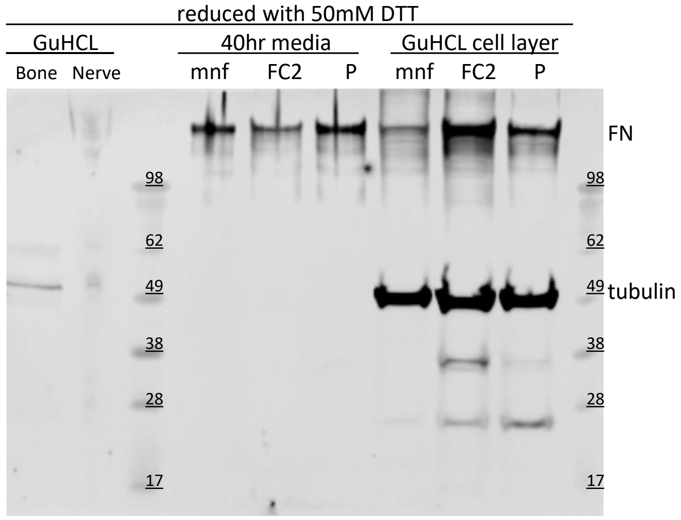
reduced with 50mM DTT
GuHCL
Bone
Nerve
40hr media
mnf
FC2
P
GuHCL cell layer
mnf
FC2
P
FN
98
62
49
38
28
17
tubulin
