# Supplementary material for: An ancestral 10-bp repeat expansion in VWA1 causes recessive hereditary motor neuropathy
Source: Brain. 2021 Jan 18;144(2):584–600. doi: 10.1093/brain/awaa420 (PMC8263055; doi:10.1093/brain/awaa420)
Supplement: awaa420_Supplementary_Data [file awaa420_supplementary_data.zip › brain-2020-01257-File015.pdf]

Genomics England Research Consortium

John C. Ambrose[1]; Prabhu Arumugam[1]; Emma L. Baple[1]; Marta Bleda[1]; Freya Boardman-Pretty[1,2]; Jeanne M. Boissiere[1]; Christopher R. Boustred[1]; Helen Brittain[1]; Mark J. Caulfield[1,2]; Georgia C. Chan[1]; Clare E. H. Craig[1]; Louise C. Daugherty[1]; Anna de Burca[1]; Andrew Devereau[1]; Greg Elgar[1,2]; Rebecca E. Foulger[1]; Tom Fowler[1]; Pedro Furió-Tarí[1]; Adam Giess[1]; Joanne M. Hackett[1]; Dina Halai[1]; Angela Hamblin[1]; Shirley Henderson[1,2]; James E. Holman[1]; Tim J. P. Hubbard[1]; Kristina ibáñez[1,2]; Rob Jackson[1]; Louise J. Jones[1,2]; Dalia Kasperaviciute[1,2]; Melis Kayikci[1]; Athanasios Kousathanas [1]; Lea Lahnstein[1]; Kay Lawson[1]; Sarah E. A. Leigh[1]; Ivonne U. S. Leong[1]; Javier F. Lopez[1]; Fiona Maleady-Crowe[1]; Joanne Mason[1]; Ellen M. McDonagh[1,2]; Loukas Moutsianas[1,2]; Michael Mueller[1,2]; Nirupa Murugaesu[1]; Anna C. Need[1,2]; Peter O'Donovan[1]; Chris A. Odhams[1]; Andrea Orioli [1]; Christine Patch[1,2]; Mariana Buongermino Pereira[1]; Daniel Perez-Gil[1]; Dimitris Polychronopoulos[1]; John Pullinger[1]; Tahrima Rahim[1]; Augusto Rendon[1]; Pablo Riesgo-Ferreiro[1]; Tim Rogers[1]; Mina Ryten[1]; Kevin Savage[1]; Kushmita Sawant[1]; Richard H. Scott[1]; Afshan Siddiq[1]; Alexander Sieghart[1]; Damian Smedley[1,2]; Katherine R. Smith[1,2]; Samuel C. Smith [1]; Alona Sosinsky[1,2]; William Spooner[1]; Helen E. Stevens[1]; Alexander Stuckey[1]; Razvan Sultana[1]; Mélanie Tanguy[1]; Ellen R. A. Thomas[1,2]; Simon R. Thompson[1]; Carolyn Tregidgo[1]; Arianna Tucci[1,2]; Emma Walsh[1]; Sarah A. Watters[1]; Matthew J. Welland[1]; Eleanor Williams[1]; Katarzyna Witkowska[1,2]; Suzanne M. Wood[1,2]; Magdalena Zarowiecki[1].

1. Genomics England, London, UK

2. William Harvey Research Institute, Queen Mary University of London, London, EC1M 6BQ, UK.

Details are correct at time of writing but regularly updated at www.genomicsengland.co.uk/about-gecip/publications.
