# Supplementary material for: An ancestral 10-bp repeat expansion in VWA1 causes recessive hereditary motor neuropathy
Source: Brain. 2021 Jan 18;144(2):584–600. doi: 10.1093/brain/awaa420 (PMC8263055; doi:10.1093/brain/awaa420)
Supplement: awaa420_Supplementary_Data [file awaa420_supplementary_data.zip › OP-BRAI200424_PECorr_AttachmentsFolder_VWA1_manuscript_supplementary[AU].pdf]

Supplementary Material for "An ancestral 10bp repeat expansion in *VWA1* causes recessive hereditary motor neuropathy"

Supplementary Figures

Supplementary Figure 1: Gene expression data from GTEx
Supplementary Figure 2: Sanger sequencing and phasing variants for Family 3
Supplementary Figure 3: Nanopore sequencing in Family 8
Supplementary Figure 4: Results of autozygosity mapping in Family 13
Supplementary Figure 5. Clinical features of youngest affected individual from Family 13
Supplementary Figure 6: Muscle MRI images
Supplementary Figure 7: Knockdown of zebrafish *vwa1*
Supplementary Figure 8: Neuronal defects observed through the development of spinal motor neurons in the *vwa1* zebrafish model
Supplementary Figure 9: Neuromuscular junction defects observed in *vwa1* zebrafish model
Supplementary Figure 10: Zebrafish *vwa1* is required for jaw joints and patterns the ventral cartilage
Supplementary Figure 11: *vwa1* knockout had no impact on the first neuronal wiring
Supplementary Figure 12: Reduced locomotor behaviour in zebrafish *vwa1* model
Supplementary Figure 13: Customised view of the *VWA1* locus from UCSC genome browser

Supplementary Tables

Supplementary Table 1: Variant details
Supplementary Table 2: Size of shared founder haplotypes used to estimate age of p.G25Rfs*74
Supplementary Table 3: Mutation age in generations with 95% confidence intervals
Supplementary Table 4: Mutation age in years
Supplementary Table 5: Candidate homozygous regions detected in Family 13
Supplementary Table 6: More detailed clinical information for all families
Supplementary Table 7: Global population allele frequencies for the common pathogenic mutations responsible for autosomal-recessive CMT/SMA

Supplementary Methods

- **Variant filtering strategies for data from the 100KGP and estimating allele frequency for founder mutation**
- **Allele-specific PCR for phasing variants in Family 3**
- **Nanopore analysis for phasing variants in Family 8**
- **Preliminary data processing for haplotype analysis**
- **Detailed description of autozygosity analysis in Family 13**

Supplementary Case Reports

Supplementary Videos 1-8

Supplementary Discussion

Supplementary References

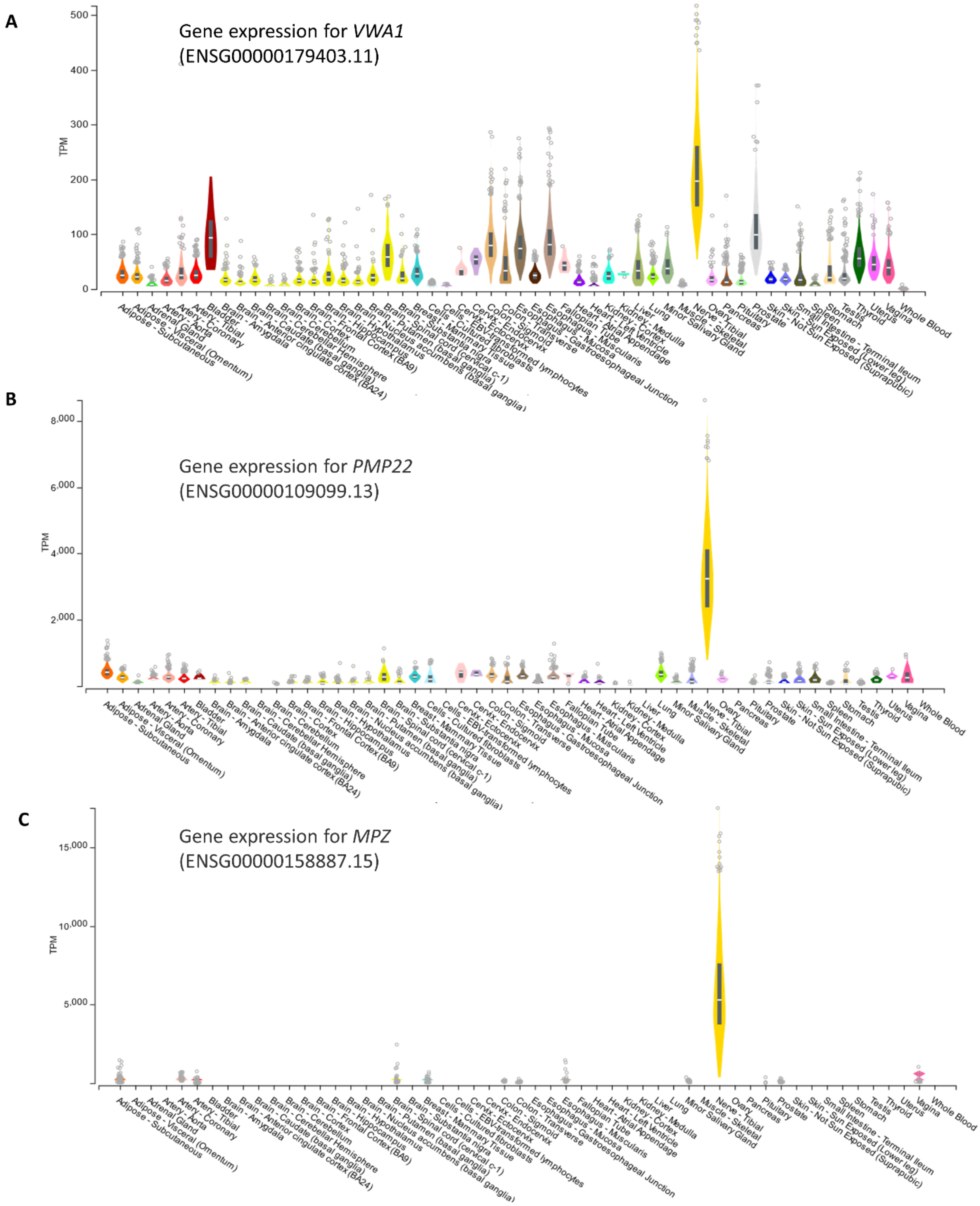

Supplementary Figure 1: Gene expression data from the GTEx resource (https://gtexportal.org). Expression across 54 different tissues or cell lines is shown. Relative

expression of each of *VWA1* (A), *PMP22* (B) and *MPZ* (B) is highest in the tibial nerve compared with other tissues. *PMP22* (MIM *601097) and *MPZ* (MIM *159440) are both well-known disease genes for Charcot-Marie Tooth disease. For *VWA1*, the median transcripts per million (TPM) = 196.9 (n = 619).

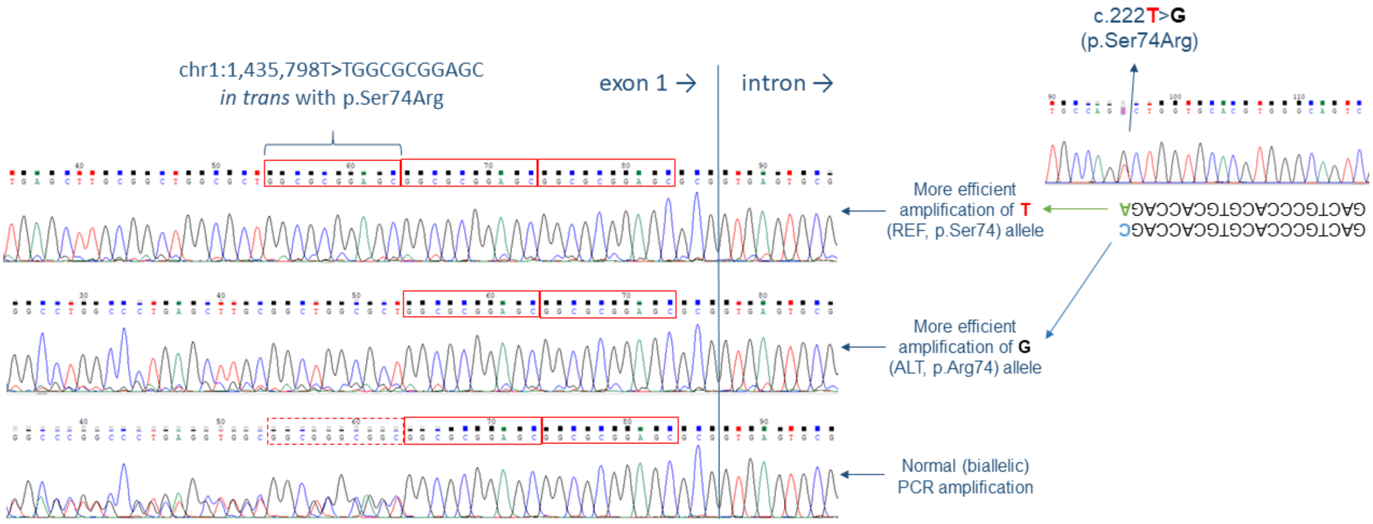

Supplementary Figure 2: Sanger sequencing electropherograms and summary of the allele-specific PCR strategy used to phase *VWA1* variants for Family 3. The repeat expansion was only seen where the reverse primer complementary to the T (ref) allele was used, indicating that the two variants lie *in trans*. The trace generated using normal (biallelic) PCR amplification is also provided for comparison.

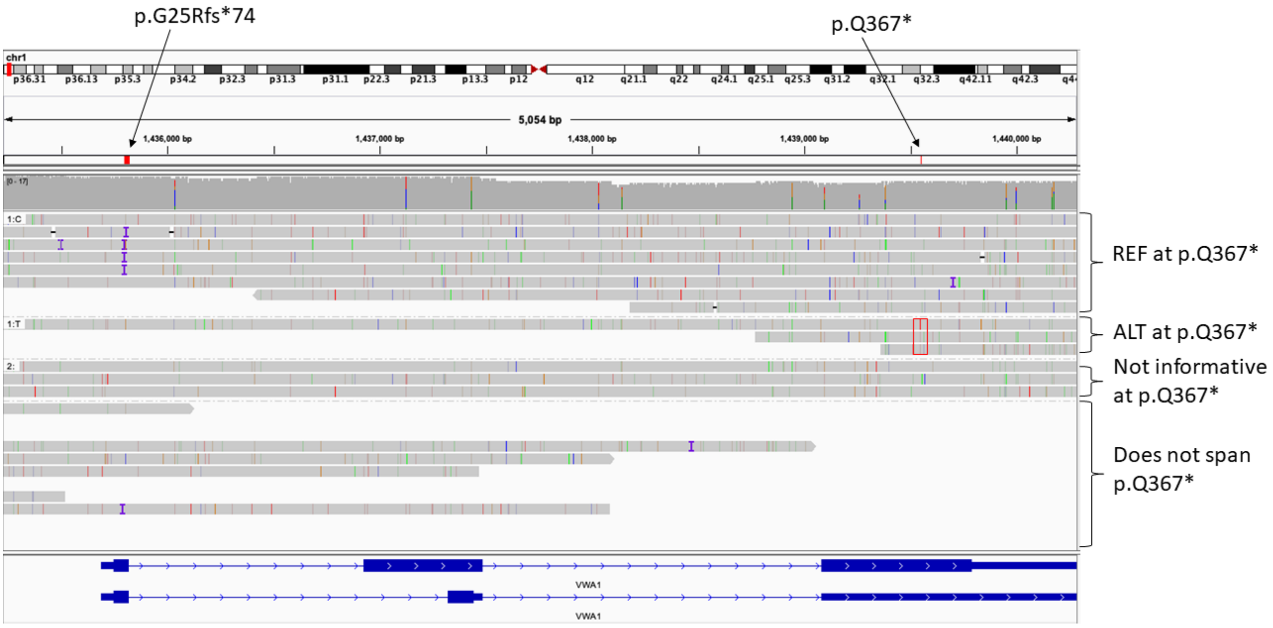

Supplementary Figure 3: Nanopore sequencing in Family 8 suggests p.G25Rfs*74 and p.Q367* variants are *in trans*. Reads were grouped with respect to the base seen at the p.Q367* variant locus, as indicated. The four reads supporting the insertion in exon 1 all have the reference base at the site of the p.Q367* variant. Only indels >8bp are shown. Although two reads (1 and 6) are discordant, we note these have a higher than average gap-compressed per-base sequence divergence (12.7% and 15.3%) as calculated by minimap2. The mean gap-compressed per-base sequence divergence of all aligned reads was 8.9%.

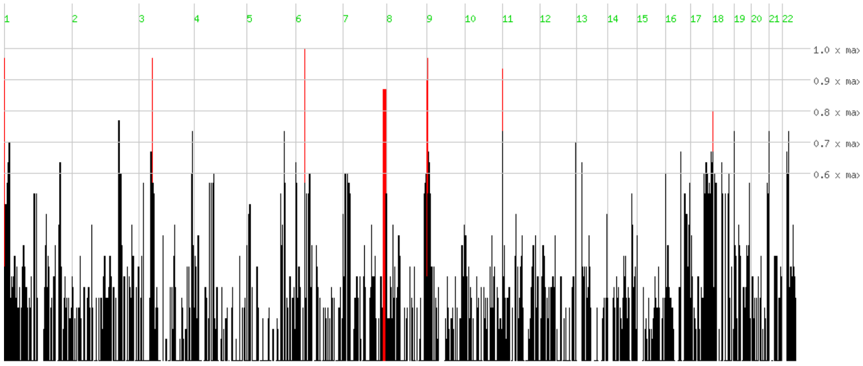

Supplementary Figure 4: Results of autozygosity mapping in Family 13. The vcf files from exome sequencing of the proband and the affected uncle were analysed using www.homozygositymapper.org. Although both affected individuals were male, the pedigree structure (Fig. 1A) makes an X-linked mode of inheritance unlikely.

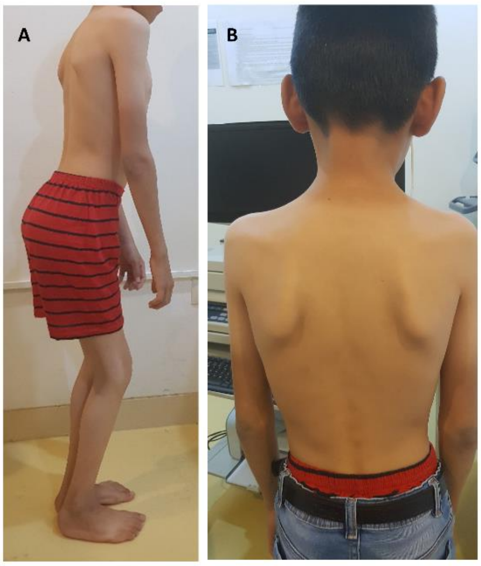

Supplementary Figure 5: Clinical features of the youngest affected individual from Family 13. A) Side view of the proband showing bilateral hip and knee flexion contractures with forward stood posture. Reduced muscle bulk in the proximal>distal lower limbs can be seen. B) Image of the proband showing scapular winging.

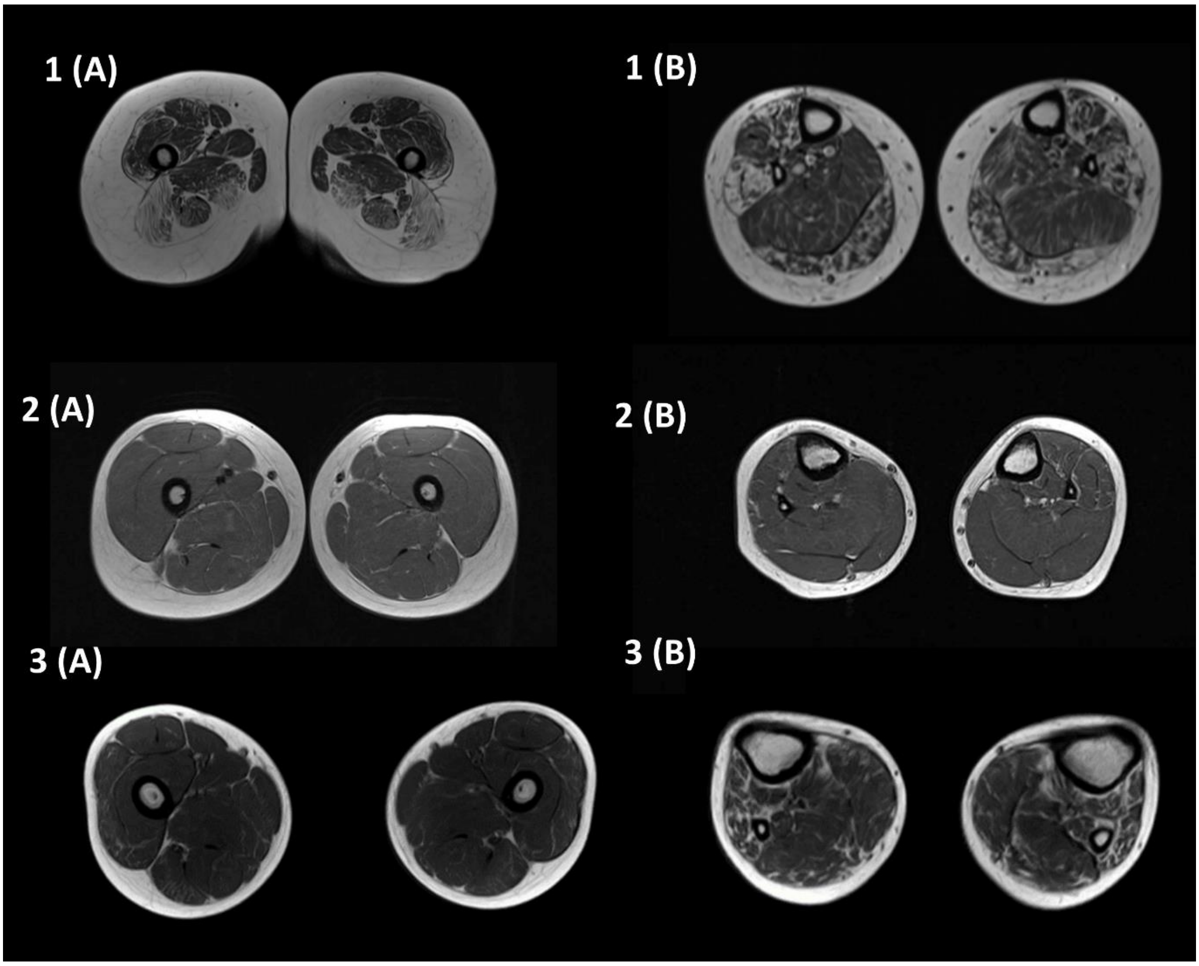

Supplementary Figure 6: Muscle MRI images (T1-weighted sequences) from patients in Families 1, 11 and 14. 1 (A)- Family 1 MRI of the proximal lower limb muscles showing fairly uniform bilateral symmetric fatty replacement with more severe fatty infiltration of the vastus lateralis. 1 (B) - Family 1 MRI of the distal lower limb showing severe fatty infiltration of the peronei muscles and tibialis anterior, with relative sparing of the extensor digitorum longus. Fatty infiltration of the medial and lateral gastrocnemius, and to a lesser extent of the soleus in the posterior compartment. 2 (A,B) Family 11 MRI of the thighs and distal lower limbs showing subtly increased signal intensity in the tibialis anterior muscles. 3 (A) - Family 14 thigh MRI showing mild fatty replacement in the vastus lateralis muscles bilaterally. 3 (B) - MRI of the lower leg showing some fatty infiltration, which is much more severe in the anterior compartment (tibialis anterior, extensor digitorum longus, peronei).

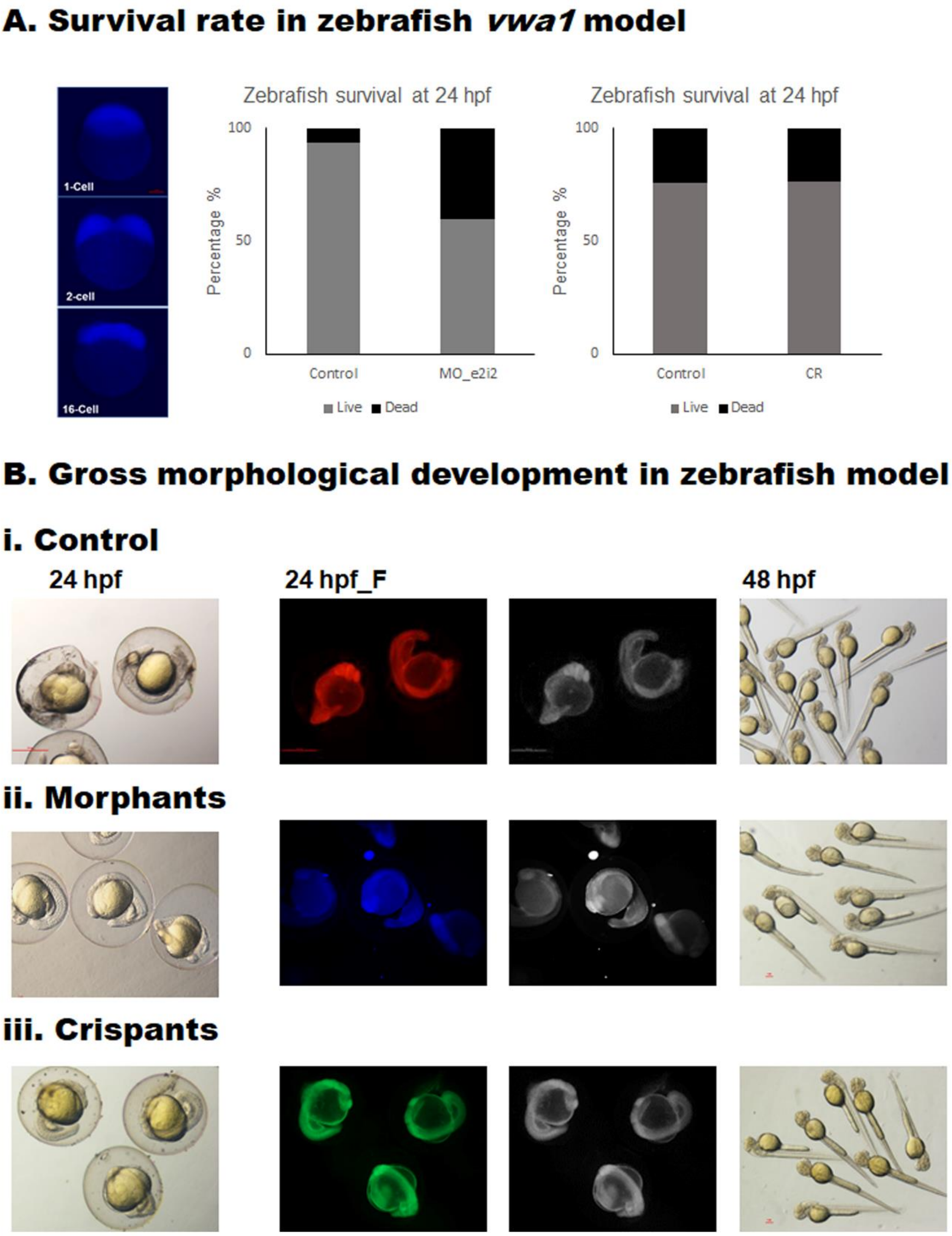

Supplementary Figure 7: Zebrafish *vwa1* model. A. Injected zebrafish embryo at 1-cell stage with *vwa1* MO (blue-emitting fluorescent tag at 3') targeting exon2 splice site. Images of cell divisions at 1, 2, and 16-cells stages using fluorescent imaging with Zeiss Lumar 12 microscope at 100X magnification. *vwa1* knockdown (n=72) had a negative effect on survival rate

compared to control group (n=93) and *vwa1* knockout (n=168) showed a similar survival rate as the control group (n=161). B. Gross morphological development of the zebrafish examined groups. The examined groups exhibited normal gross morphology comparable to the control group, in terms of body length and shape.

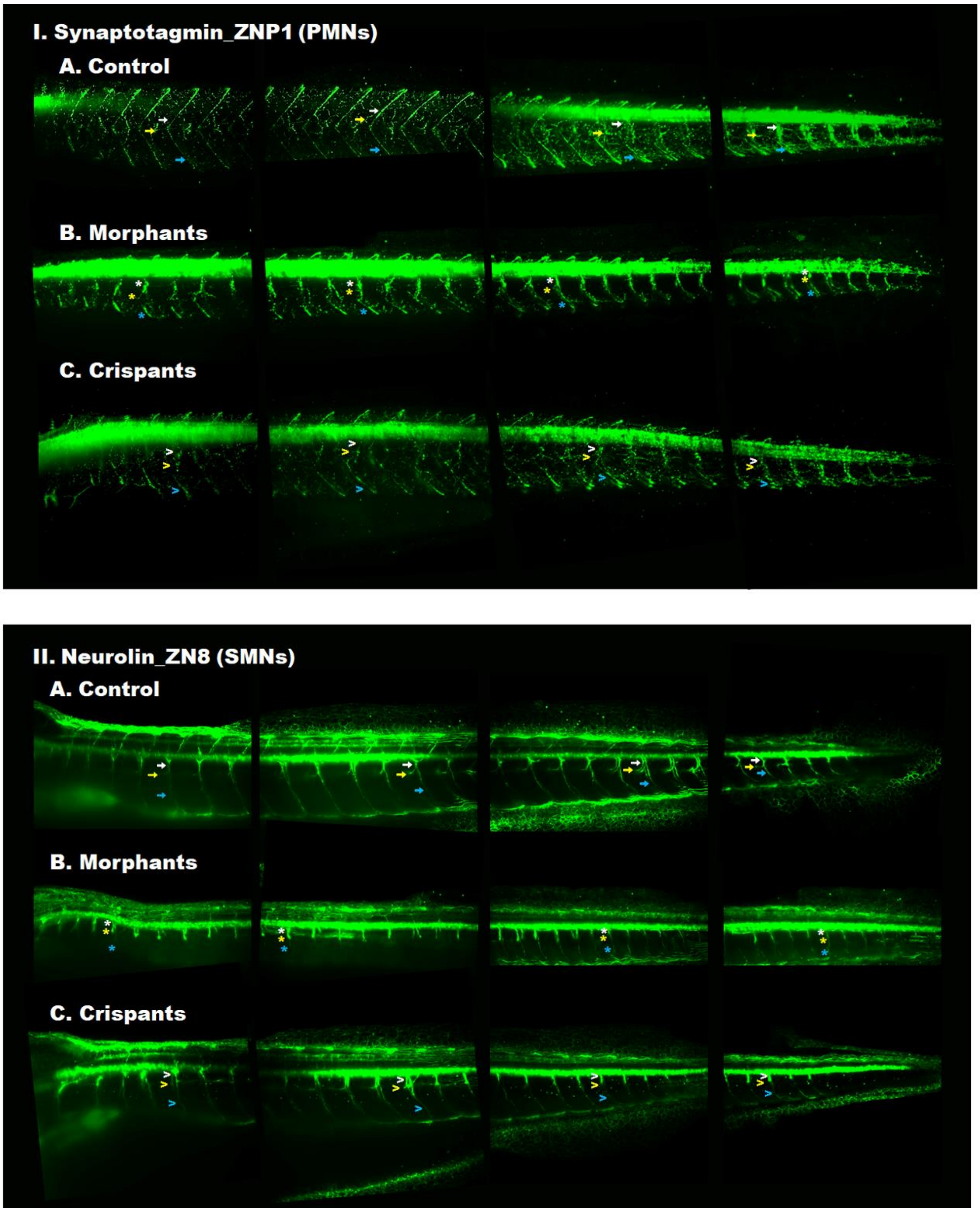

Supplementary Figure 8: Neuronal defects observed through the development of spinal motor neurons in the *vwa1* zebrafish model. To visualize primary motor neurons (PMNs) and

secondary (SMNs), fixed zebrafish were labelled with synaptotagmin (znp1 antibody) and neurolin (zn8 antibody) respectively. Images are lateral view of whole-mount immunofluorescence performed to label axons from the spinal cord at 72 hpf zebrafish embryos. A. Control embryos, rostral (RoP) (white), middle (MiP) (yellow), and caudal (CaP) (blue) MN axons innervated fast muscle fibers located in the frontal, middle, dorsal, and ventral region of the zebrafish, respectively. B. Morphants showed significantly decreased axonal branching with some shorter MN axons (asterisks) (I.B); most of the axons of MiP SMNs (yellow) were absent (II.B) and CaP SMNs (blue) were severely impacted (II.B) and in some SMNs, an early truncation of SMN axons was observed. C. Crispants displayed a subtle PMNs morphology (I.C); while the SMN axons MiP MNs (yellow) were severely impacted and in some SMNs (II.C) and shorter CaP MNs (blue) in some SMNs (II.C). Representative images of 4 sections through the zebrafish body of z-stack compilations of maximal projections of whole mount zebrafish at 40X magnification using Zeiss Lighsheet Z1 microscope.

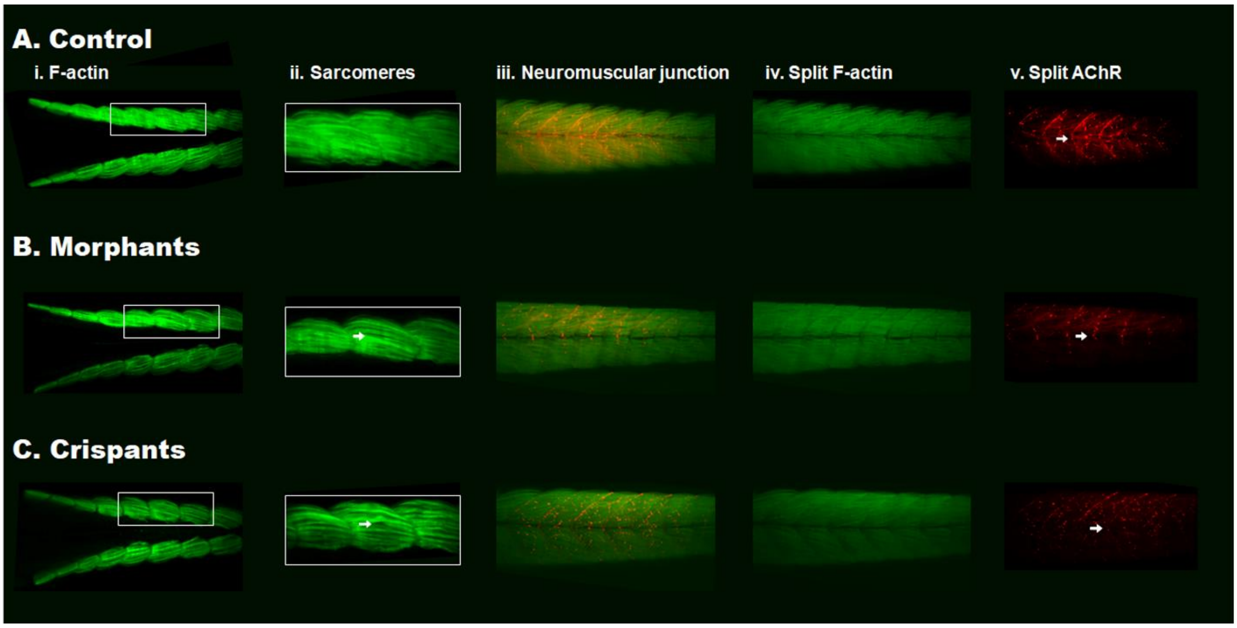

Supplementary Figure 9: Neuromuscular junction (NMJ) defects observed in *vwa1* zebrafish model. Analysis of muscle fiber and neuromuscular junction formation using Phalloidin (Green, F-actin) and alpha-Bungarotoxin (Red, AChR). A. Control embryos displayed densely packed muscle fibers with organized assembly and bundling of the sarcomeres, dorsal view of the stained somites at 72 hpf (i) with a subset of sarcomeres 5-7 (ii). Staining of the NMJs showed an organized synaptic formation through the developing somites (iii-v). B-C. Zebrafish *vwa1* Morphants and Crispants revealed decreased muscle integrity with less abundant

myofibers and significantly reduced NMJ arborization. Multiple image stacks were collected using 1µm z-steps through the entire thickness of the embryo samples. Representative images of 4 sections through the zebrafish body of z-stack compilations of maximal projections of whole mount zebrafish at 40X magnification using Zeiss Lightsheet Z1 microscope.

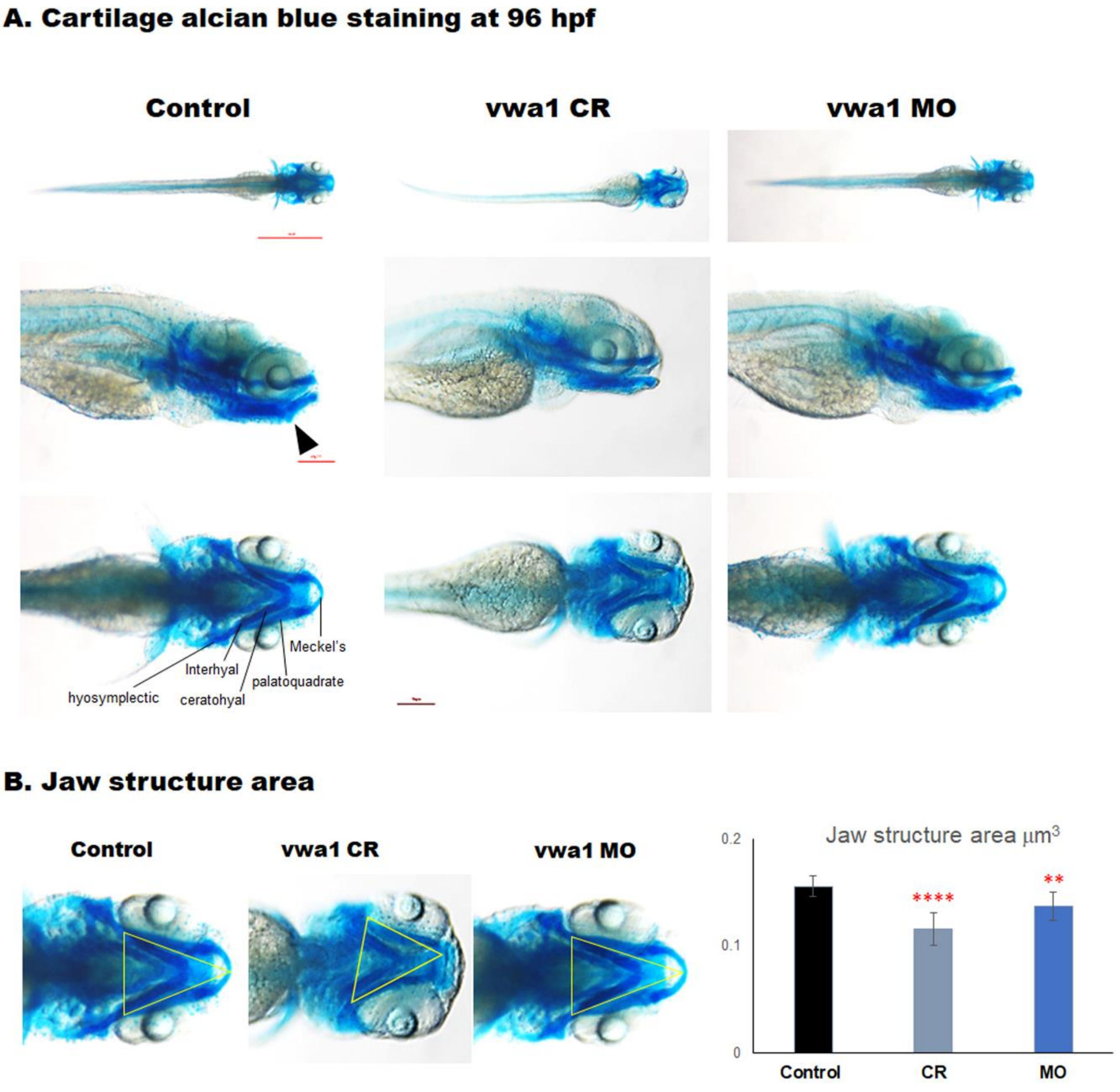

Supplementary Figure 10: Zebrafish *vwa1* is required for jaw joints and patterns the ventral cartilage. A) Representative images of ventral view for the examined groups at 96 hpf. Flat mounts of stained cartilage with alcian blue. Distinct jaw elements are visible in control, *vwa1* crispant and morphant groups. The knockout model demonstrated a noticeable abnormal development of the cartilage at the branchial arches and the jaw. The structure of the arches Meckel's and palatoquadrate became irregular in the *vwa1* zebrafish model. B) Measurement of jaw structure area from the Meckel's to Interhyal structure showed a significant reduction

in the area for the crispants with a mean of 0.116µm^3 (n=16, p <0.0001) and for the morphants with a mean of 0.137µm^3 (n=8, p=0.0075) when compared to control group with a mean of 0.156µm^3 (n=15). Zebrafish imaging using Lumar 12 microscope at a magnification of 100X (scale bar 10 µm). One way ANOVA test was used for statistical analysis (GraphPad software 8.0).

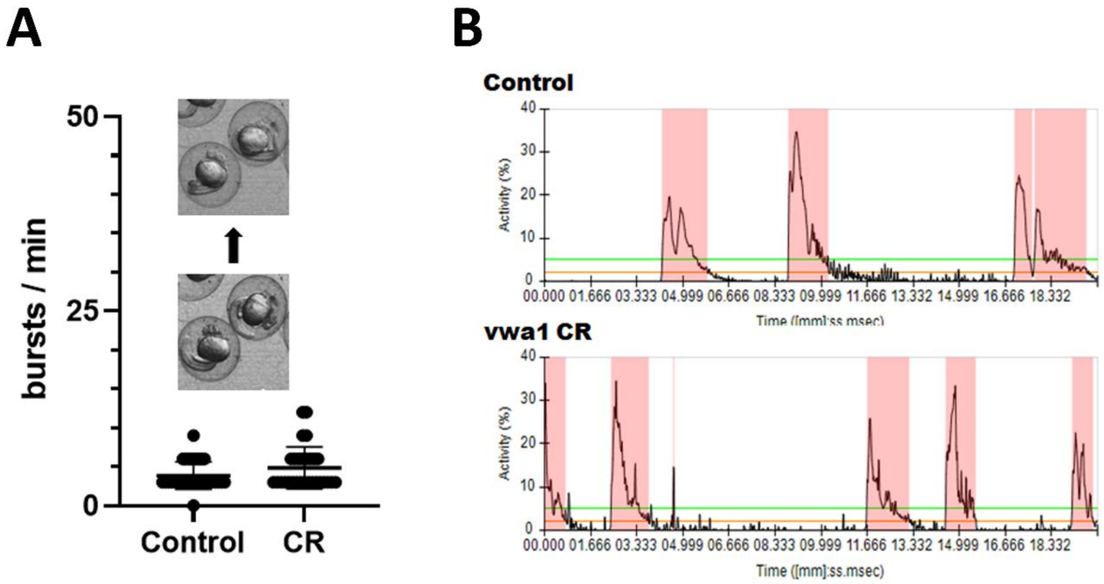

Supplementary Figure 11: *vwa1* knockout had no impact on the first neuronal wiring. Zebrafish *vwa1* crispants had no impact on spontaneous head and tail coils that start at 24 hours postfertilization (hpf). The mean tail coils was at 4.86 bursts per minute compared to the control group mean at 3.87. A. Locomotive behaviour of zebrafish embryos after *vwa1* crispr injections assessed by spontaneous tail flicking and embryos movements that were measured by embryo burst count per minute and plotted for the mean. Control n=31 and crispants n=29. Statistical analysis was performed with t-test using GraphPad prism (8.0). B. Representative activity chart of zebrafish embryo at 24 hpf measuring movements through time for the zebrafish examined groups.

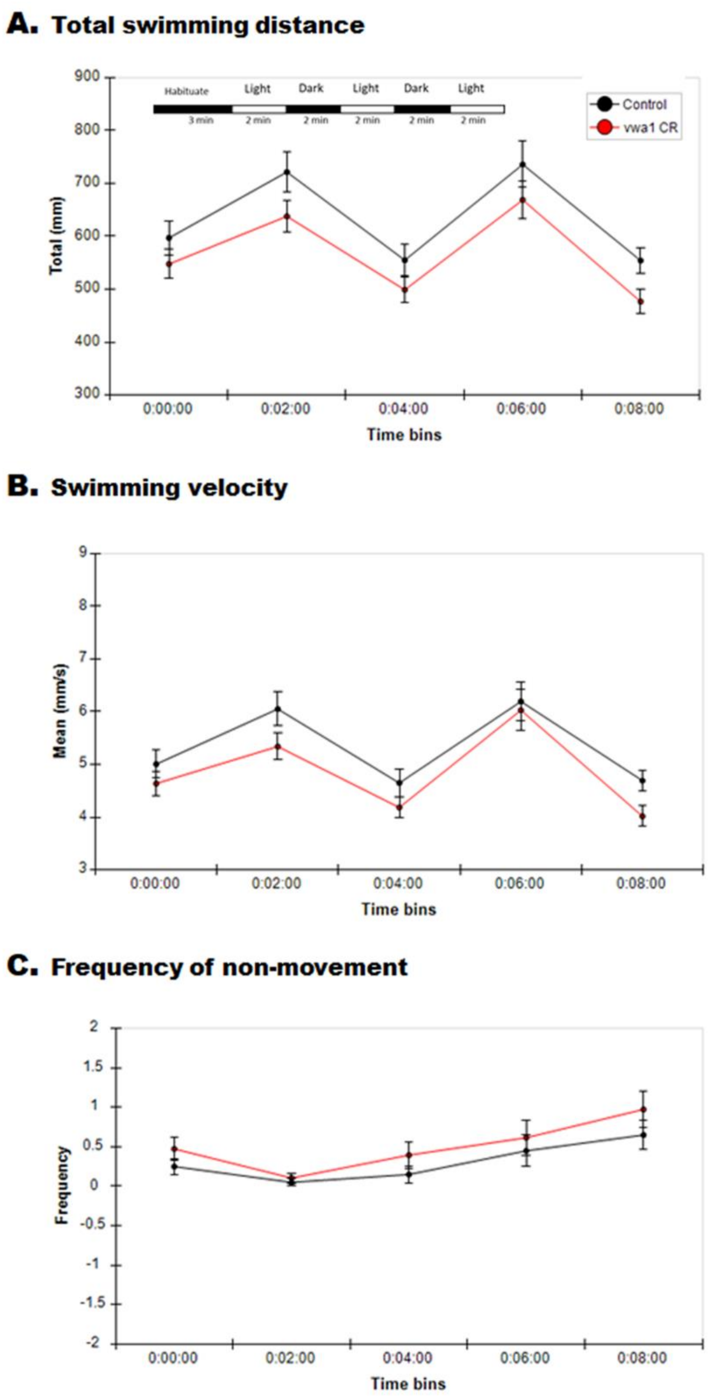

Supplementary Figure 12: Reduced locomotor behaviour in zebrafish *vwa1* model. Analysis of zebrafish *vwa1* CRISPR KO model (n=20) showed A) reduced total distance moved over time B) reduced velocity and C) increased frequency of non-movements from centre point when compared to control group (n=10). The analysed parameters were assessed by locomotive behaviour recording through the presentation of 2 minutes light-dark cycles over a time interval

of 10 minutes). Statistical analysis was performed with t-test using GraphPad prism (version 8.0).

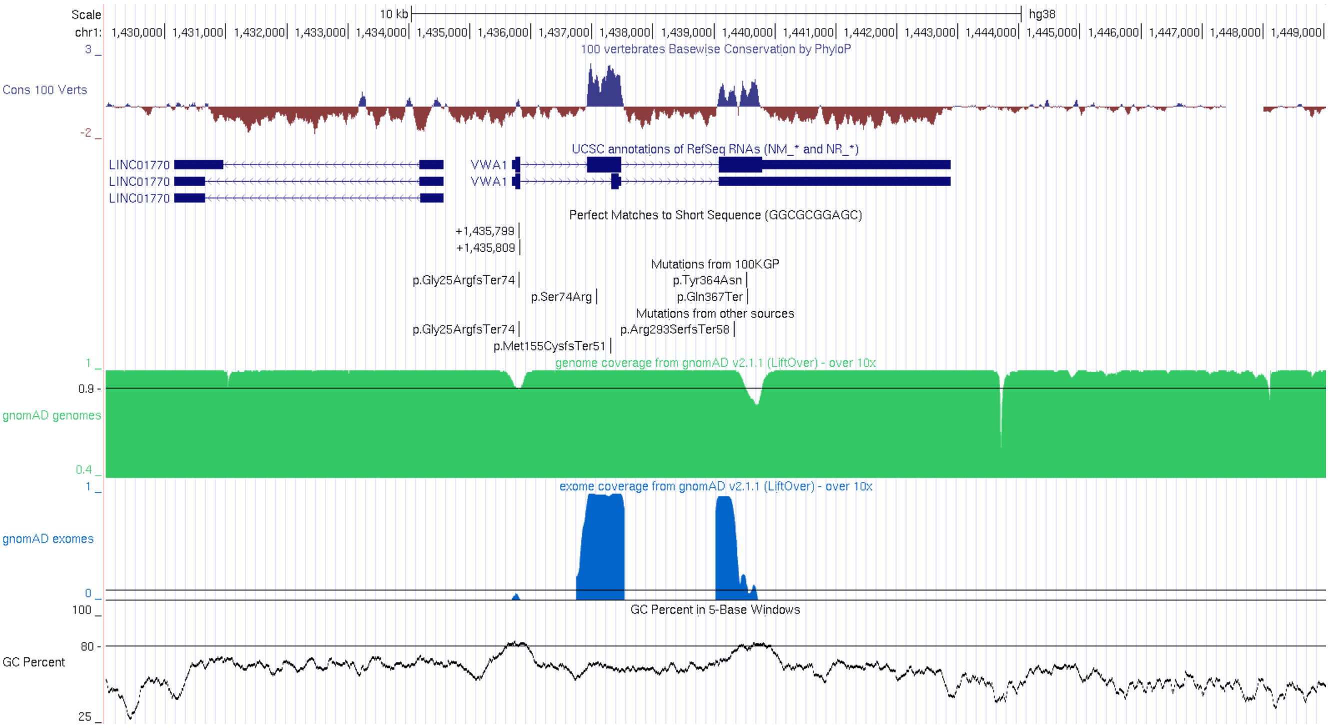

Supplementary Figure 13: Customised view of the *VWA1* locus from UCSC genome browser showing conservation (PhyloP), RefSeq gene annotations, the position of the 10bp repeat in exon 1 and the variants described in this study. Custom tracks show the percentage of genomes (green) and exomes (blue) in gnomAD v2.1.1 for which there is at least 10x coverage, with genomic positions switched to GRCh38 using the LiftOver tool. The GC content of this locus is shown using a 16 pixel smoothing window. An interactive version is available: https://genome.ucsc.edu/s/AlistairP/VWA1_GCcontent_coverage.

Supplementary Table 1: Variant details including genomic and cDNA annotations, predicted protein changes, variant allele frequencies from across a number of databases and scoring based on ACMG criteria (Richards *et al.*, 2015). VUS, variant of uncertain significance. For data from gnomAD, European refers to non-Finnish Europeans.

**Variant annotation**	GRCh38/hg38 position (DNA change)	chr1:1,435,798 (T>TGGCGCGGAGC)	chr1:1,437,075 (T>G)	chr1:1,437,308 (GC>G)	chr1:1,439,326 (AG>A)	chr1:1,439,539 (T>A)	chr1:1,439,548 (C>T)
	cDNA change (NM_022834.5)	c.62_71dup10	c.222T>G	c.462delC	c.879delG	c.1090T>A	c.1099C>T
	Protein change	p.G25Rfs*74	p.S74R	p.M155Cfs*51	p.R293Sfs*58	p.Y364N	p.Q367*
	dbSNP ID	rs1371393163	rs768379861	rs760281341	rs746212067	NA	NA
	Variant seen in family	1-12, 14-15	3	13	15	10	8
**Allele frequencies (PM2)**	gnomAD v3 (highest subpopulation)	0.063%, 89/140,632 (0.091%, 58/63,526 in Europeans)	0.003%, 4/143,318 (0.005%, 3/64,576 in Europeans)	absent	0.007%, 10/143,112 (0.030%, 1/3,318 in Ashkenazi Jewish	absent	absent
	gnomAD v2.1.1 (highest subpopulation)	0.033%, 15/45,050 (0.070%, 6/8,608 in Africans)	0.004%, 9/246,020 (0.008%, 9/110,178 in Europeans)	0.002%, 5/242,458 (0.010%, 3/30,072 in South Asians)	0.006%, 13/220,412 (0.013%, 13/98,184 in European)	absent	absent
	Frequency in 100KGP (B38 aggregate vcf file dated 28th Feb 2019 based on ISAAC calls)	0.107%, 127/118,928<br><br>or 0.094% (112/118,908) if families described here are removed	0.008%, 10/118,928<br><br>or 0.008% (9/118,926) if family 3 removed	0.003%, 4/118,928	absent	absent (genomes for family 10 only available in April/May 2019)	0.001% (1/118,928)<br><br>or absent if family 8 removed
	Iranome	absent	absent	absent	absent	absent	absent
	GME Variome	absent	absent	absent	absent	absent	absent
	CentoGene internal database	0.019%, 26/136,000	0.001%, 1/136,000	0.003%, 4/136,000	absent	absent	absent
	GeneDx exome database	0.087%, 188/215,908 (0.112%, 114/101,380 in Europeans)	0.007%, 15/209,288	absent	0.006%, 12/212,794	absent	absent
***In silico* predictions (PP3)**	CADD	24	23	10.31	22.2	28.7	40
	Polyphen	NA	Probably damaging	NA	NA	Probably damaging	NA
	SIFT	NA	Deleterious	NA	NA	Deleterious	NA
	MutationTaster	Disease causing	Disease causing	Disease causing	Disease causing	Disease causing	Disease causing
	Predicted ddG (kcal/mol)	NA	+24 (destabilising)	NA	NA	+10 (destabilising)	NA
**Additional ACMG criteria and classification**	Observed *in trans* with pathogenic variant? (PM3)	Yes p.Q367* in family 8	Yes (p.G25Rfs*74 in family 3)	No	Yes (p.G25Rfs*74 in family 15)	Yes (p.G25Rfs*74 in family 10)	Yes (p.G25Rfs*74 in family 8)
	Support from cosegregation within family (PP1)	No (twins in family 12 are MZ and in most families the unaffected siblings have not been tested	No	Yes (homozygous in both proband and in affected uncle)	Yes (although just a single unaffected sibling)	No	No
	Support from enrichment of homozygotes in 100KGP (PS4)	Yes	No	No	No	No	No
	Null variant (nonsense, frameshift, canonical splice site) (PVS1)	Yes	No	Yes	Yes	No	Yes (19% of mature protein is removed)
	Effect on transcript/protein (PS3)	Partial NMD and no detectable protein	NA	NA	NA	NA	NA
	Overall classification	Pathogenic (PM2, PP3, PM3, PS4, PVS1, PS3)	VUS (PM2, PP3, PM3)	Pathogenic (PM2, PP3, PP1, PVS1)	Pathogenic (PM2, PP3, PP1, PVS1)	VUS (PM2, PP3, PM3)	Pathogenic (PM2, PP3, PM3, PVS1)

Supplementary Table 2: Size of shared founder haplotypes used to estimate age of p.G25Rfs*74. Genomic positions are given based on hg19 in order to be compatible with the online mutation dating application (https://shiny.wehi.edu.au/rafehi.h/mutation-dating/).

ID	start (bp)	end (bp)	size (kb)	start (cM)	end (cM)
Family 1	1,103,542	1519,068	415.5	2.78	3.25
Family 2	1,106,473	1519,068	412.6	2.81	3.25
Family 4	1,103,542	1575,046	471.5	2.78	3.36
Family 5	1,158,070	1575,046	417.0	2.94	3.36
Family 6	1,124,750	1432,485	307.7	2.91	3.20
Family 7	1,103,542	1519,068	415.5	2.78	3.25
Family 9	1,155,804	1,378,513	222.7	2.93	3.10

Supplementary Table 3: Mutation age in generations with 95% confidence intervals. Genealogies are rarely independent, and unless there is information to indicate otherwise, genealogies should be assumed to be correlated.

	Generations	CI (lower)	CI (upper)
Assuming an 'independent' genealogy:	399.5	235.2	683.2
Assuming a 'correlated' genealogy:	311.0	106.9	974.6

Supplementary Table 4: Mutation age in years (assuming one generation = 25 years) with 95% confidence intervals.

	Years	CI (lower)	CI (upper)
Assuming an 'independent' genealogy:	10,000	5,875	17,075
Assuming a 'correlated' genealogy:	7,775	2,675	24,375

Supplementary Table 5: Candidate homozygous regions detected in Family 13 using www.homozygositymapper.org. Positions are based on GRCh38/hg38. *Region contains *VWA1*.

Score	Chr	Start	End	Size
30	6	32,555,062	32,558,138	3,076
29	1	137,825	1,646,565	1,508,740*
29	3	46,743,865	48,588,247	1,844,382
29	9	4,722,685	4,944,758	222,073
28	11	792,692	824,805	32,113
27	9	417,990	446,223	28,233
26	7	144,548,450	154,853,696	10,305,246
24	18	49,034	49,824	790

Supplementary Table 6: More detailed clinical information for all families. *Available as separate xlsx file.*

Supplementary Table 7: Global population allele frequencies for common pathogenic variants responsible for autosomal-recessive CMT/SMA. Frequencies are reported as allele counts and are from gnomAD v.3. Although the Table does not include structural variants we note there is also a disease causing exon 7-8 deletion in *SMN1* that is relatively common in Europeans (Vijzelaar *et al.*, 2019). Adapted from Supplementary Table 3 published by Cortese and colleagues (Cortese *et al.*, 2020). Notes column summarises technical aspects that are speculated to have delayed gene discovery.

Gene (transcript)	HGVS variant	Allele freq.	Year gene associated with disease (reference)	Notes
SORD (NM_003104.6)	c.757delG; p.A253Qfs*27	623/142,588	2020 (Cortese *et al.*, 2020)	Highly homologous pseudogene harbouring same polyA contraction; nested-PCR required for validation
FIG4 (NM_014845.5)	c.122T>C (p.I41T)	164/143,266	2007 (Chow *et al.*, 2007)	NA
SH3TC2 (NM_024577.4)	c.2860C>T; p.R954*	94/143,270	2003 (Senderek *et al.*, 2003)	NA
VWA1 (NM_022834.5)	c.62_71dup; p.G25Rfs*74	89/140,632	2020 (present study)	High GC content resulting in limited coverage upon exome sequencing; expansion of 2x10bp repeat
GDAP1 (NM_018972.4)	c.487C>T; p.Q163*	21/143,188	2002 (Cuesta *et al.*, 2002)	NA
IGHMBP2 (NM_002180.2)	c.2911_2912del; p.R971Efs*4	25/143,316	2001 (DSMA1) (Grohmann *et al.*, 2001) and 2014 (CMT2S) (Cottenie *et al.*, 2014)	NA
SMN1 (NM_000344.3)	c.5C>G; p.A2G	7/1,230	1996 (Parsons *et al.*, 1996)	High GC content and SegDup region
FDG4 (NM_139241.3)	c.991delC; p.Q443Rfs*9	5/143,280	2007 (Delague *et al.*, 2007)	NA

Supplementary Methods

Variant filtering strategies for data from the 100KGP and estimating allele frequency for founder mutation

For Families 1-10, genome sequencing was performed as part of the 100K Genomes Project (100KGP). This project is a national genome sequencing initiative delivered jointly by the UK's National Health Service and Genomics England, a publicly run company owned by the Department of Health and Social Care. More information about this project is available at https://figshare.com/articles/GenomicEnglandProtocol_pdf /4530893/5). All research-based

analyses on this data is performed within Genomics England's secure research environment (https://re.extge.co.uk/ovd). We searched for biallelic *VWA1* variants in this resource using five different strategies:

1. An initial search was simply using the tiering data table that is available in the Labkey application. The pipeline used for the clinical tiering process is based on vcf files that have been generated using Platypus software (Rimmer *et al.*, 2014). Variants that are rare, potentially protein disrupting and that involve known genes are prioritised as TIER1 or TIER2, depending on whether they are i) *de novo* or ii) are likely to result in loss of function. Known genes are defined as those listed in PanelApp, an online database which crowdsources expert knowledge to help establish consensus for diagnostic-grade panels (Martin *et al.*, 2019). Other variants which fit in with a plausible mode of inheritance based on the pedigree structure, but which do not fall in the set of PanelApp genes linked to the presenting condition, are listed as TIER3. Using this tiering table, we searched for all TIER3 variants in *VWA1* that fitted a biallelic mode of inheritance. This approach identified Families 1-3.

2. A second approach searched specifically for the p.G25Rfs*74 founder mutation using a bcftools query of aggregated gvcf files that had been called using ISAAC software (Illumina). This aggregate file contains data on 59,464 participants (both affected and unaffected individuals) and was created in February 2019, so only contains genomes available at that time. This approach identified Families 4-7; in all of these families the affected proband was homozygous for the founder mutation. For these individuals, the candidate variant had been missed by the clinical tiering pipeline due to the Platypus call being of low confidence ("badReads") which led to the variant being filtered out at the QC stage. The command lines used are shown below:

```
module load bcftools/1.9

bcftools query -f'[%CHROM:%POS %REF %ALT %SAMPLE %GQ %GT\n]'
-i'GT="alt"                    '                    -r                    chr1:1435798
gel_data_resources/main_programme/aggregated_illumina_gvcf/
GRCH38/20190228/data/60k_GRCH38_germline_mergedgVCF_chr1_13500
02_2702774.bcf | grep '1/1'
```

This filtering approach was also useful for estimating the allele frequency of the founder variant in the UK. By removing the final "grep '1/1'" option, we were able to identify heterozygotes as well as homozygote individuals in individuals from 100KGP. Although there were 127/118,928 chromosomes with this variant, we removed the families with affected individuals that had been ascertained due to presence of this founder mutation. This resulted in an allele frequency of 0.094% (112/118,908) which is consistent with the "European non-Finnish" frequency in gnomAD v3 which is 0.091% (58/63,526).

3. A third approach employed the shell script shown below which was submitted to Genomic England's high performance compute cluster. This script searched through individual vcf files one at a time and was designed to pull out all *VWA1* variants predicted to result in loss of function. This approach identified Families 8-9. Family 8 had been overlooked by approach 1 due to the p.Q367* variant being low confidence in the platypus vcf and was missed by approach 2 due to the compound heterozygous nature of the variants. Family 9 was overlooked by approach 1 as the founder mutation was not called by Platypus and the genome was not available at the time the aggregate file for approach 2 was generated.

```
#!/bin/bash

module load bcftools/1.9

while read -r vcf; do
	tabix -h $vcf -R regions.txt | \
	bcftools norm -m -any | \
	bcftools view -f PASS -i '(MIN(FMT/DP)>10 & MIN(FMT/GQ)>15) |
(MIN(FMT/DPI)>10 & MIN(FMT/GQ)>15)' | \
	bcftools query -f
'[%SAMPLE]\t%CHROM\t%POS\t%ID\t%REF\t%ALT\t%QUAL\t%FILTE
R\t[%GT]\t[%GQ]\t([%DP]|[%DPI])\t%INFO/CSQT\t%INFO/AF1000G\n' | \
		grep -f so_terms_LOF.txt >> VWA1_results.txt ;
done < RD_standardVCF_filepaths74180.txt
```

```
sed -i
'1s/^/SAMPLE\tCHROM\tPOS\tID\tREF\tALT\tQUAL\tFILTER\tGT\tGQ\
tDP_DPI\tCSQT\tAF1000G\n/' VWA1_results.txt
```

The "regions.txt" file contains the *VWA1* gene coordinates based on both B37 and B38 reference genomes:

```
1       1371070     1378262     (B37)
chr1    1435690     1442882     (B38)
```

The "so_terms_LOF.txt" file contains SO terms that are likely to result in loss of function:

```
splice_acceptor_variant
splice_donor_variant
stop_gained
frameshift_variant
stop_lost
start_lost
```

The "RD_standardVCF_filepaths74180.txt" file contains the filepaths to all 74,180 standard vcf files available within the 100K genomes project. These had been generated using ISAAC (Illumina)

4. We then revisited the tiering table and also reviewed monoallelic variants that fitted with an autosomal dominant mode of inheritance. Where incomplete penetrance was deemed plausible at the time of submission to 100KGP, these lists of candidate variants also includes rare heterozygous variants that had been inherited from an unaffected parent. For patients where the phenotype was in keeping with affected individuals from Families 1-9, read alignments were assessed manually by loading BAM files into IGV and viewing exon 1 of *VWA1* where the founder mutation is localised. This approach identified Family 10 which had been missed by approach 1 as the founder mutation was not called with Platypus. The compound-heterozygosity explained why the variants had been missed by approach 2. Approach 3 missed this likely diagnosis as p.Y364N

is annotated as missense so was excluded by the "so term" requirement for variants to be likely loss of function.

5. *VWA1* lies on 1p36.33 and thus is within the region deleted in 1p36 deletion syndrome, one of the most common terminal microdeletion syndromes (Heilstedt *et al.*, 2003; Giannikou *et al.*, 2012). Atypical cases of European ancestry where the 1p36 deletion is associated with peripheral neuropathy should also be tested for hemizygosity of the founder p.G25Rfs*74 variant. We therefore filtered the structural variant vcf files using the shell script below. Although this identified a patient with congenital myopathy and a 1.34Mb *de novo* deletion involving *VWA1* (data not shown), no hemizygous variants were detected on the remaining copy of this gene and thus no cases with biallelic *VWA1* variants were identified by this method. Nevertheless, many microdeletions involving this chromosome region are reported in the literature and the 1.34Mb deletion appears to include the "smallest region of overlap" (Giannikou *et al.*, 2012). Therefore an unanticipated by-product of our research is to have uncovered a case with 1p36 deletion syndrome which will be fed back through the standard clinical pipeline.

```
#!/bin/bash

module load bcftools/1.9

while read -r vcf; do
	tabix -h $vcf -R regions.txt | \
	bcftools norm -m -any | \
	bcftools query -f
'[%SAMPLE=%GT,%GQ]\t%SVTYPE\t%CHROM\t%POS\t%END\t%REF\t
%ALT\t%FILTER\t%QUAL\n' >> VWA1_SV_results.txt;
done < RD_structuralVCF_filepaths74180.txt

sed -i
'1s/^/[%SAMPLE=%GT,%GQ]\t%SVTYPE\t%CHROM\t%POS\t%END\
t%REF\t%ALT\t%FILTER\t%QUAL\n/' VWA1_SV_results.txt
```

The "RD_structuralVCF_filepaths74180.txt" file contains the filepaths to all 74,180 structural variant vcf files available within the 100K genomes project. These SV calls had been generated using a combination of Manta and Canvas software.

The "regions.txt" file is as shown above for approach 3.

Allele-specific PCR for phasing variants in Family 3

The affected individual in Family 3 was recruited to the 100KGP as a singleton and due to the patient's age, it was not feasible to collect follow-up DNA samples from the parents. The p.G25Rfs*74 and p.S74R variants identified were 1,277bp apart and thus too far apart to phase using the Illumina read-pair information. We therefore used an allele-specific PCR strategy to phase these two rare variants. After first validating the c.222T>G; p.S74R variant using primers GGACCTGATGTTCCTGCTGG, AGAAGCACGCACCGCATC and a standard Sanger sequencing protocol, we designed two reverse primers (Supplementary Fig. 2) where the final 3' base was complementary to either the T (ref) or G (alt) allele. Using one or other of these R primers, we performed PCR amplification together with a forward primer (CGATGCTCCCCTGGACGG). Amplification was done using the FastStart Taq polymerase kit with the additional GC rich additive. Annealing was at 61°C and the extension time was increased to 60 seconds. The PCR product was then purified using the *exo*I and SAP enzymes. Sanger sequencing was performed using an internal R primer (CTCCCGCAGCCTCCCAGC).

Nanopore analysis for phasing variants in Family 8

The affected individual in Family 8 had also been recruited to the 100KGP as a singleton and due to the patient's age, it was again not feasible to collect parental samples for follow-up. The p.G25Rfs*74 and p.Q367* variants identified were 3,750bp apart and thus too far apart to phase using the Illumina read-pair information. Due to the high GC content we were unsure whether long-range allele-specific PCR (as described above) would work and therefore chose to enter this patient into a Nanopore sequencing pilot study. Germline DNA (5 µg, 65 ng/µl) was depleted of low molecular weight DNA (<10 Kb) with the Short Read Eliminator kit (Circulomics, SS-100-101-01) as described by the manufacturer. After checking DNA size distribution on an Agilent Femto Pulse system, a sequencing library was generated with the Oxford Nanopore SQK-LSK109 kit, starting from 800 ng of high molecular weight-enriched DNA. In brief, DNA was subjected to end-repair/dA-tailing and adapter ligation following

manufacturer's recommendation, with the exception that after the second AmpureXP beads purification DNA was eluted for 15 minutes at 37°C in 24 µl of buffer EB. Samples were quantified with a Qubit fluorometer (Invitrogen, Q33226) and 300 ng loaded onto a PromethION R.9.4.1 flow cell according to Oxford Nanopore recommendations. Base-calling was performed with Guppy-3.2.10 in high accuracy mode. Reads passing filter were aligned to GRCh38 with Minimap2-2.17 using --secondary=no and –MD options. Aligned reads were visualised with IGV-2.5.2 using the quick consensus function.

Preliminary data processing for haplotype analysis

Genome sequencing data for the individual from Family 2 which had originally been analysed using the GRCh37 genome build was reprocessed using GRCh38. Joint variant calling was then performed within Genomics England's Research environment for all 7 individuals who were homozygous for the p.G25Rfs*74 variant. In order to minimise chances of conclusively revealing closely related individuals, analysis was limited to the first 20Mb of chromosome 1. Variant calling was with Platypus v0.8.1, using the "GCA_000001405.15_GRCh38_no_alt_analysis_set.fna" reference genome and default settings, except for "minFlank=0". The output multi-sample vcf file was then uploaded to the VEP web interface (www.ensembl.org/Tools/VEP) in order to annotate with global allele frequencies from the 1000 Genomes project. We then filtered for PASS variants and removed indels. Unlikely genotypes that disrupted large segments of identity-by-descent were reviewed using IGV v2.3.92.

Detailed description of autozygosity analysis in Family 13

For Family 13, exome sequencing was performed by Macrogene (South Korea). Genetic variants were identified using the GATK suite v4.0.4.0 according to the best-practices workflow. Briefly, variants were first called in single individuals using Haplotype Caller to generate gVCF files. Genomic VCFs were then merged using CombineGVCFs and a standard VCF representing all samples was generated using GenotypeGVCFs with a minimum variant quality of 20. The multisample vcf was then uploaded to www.homozygositymapper.org using only known variants and a coverage threshold setting of 10x. Default analysis settings were used but with the requirement for genetic homogeneity, as the two affected individuals (proband and uncle) are from the same family. All variants detected were then analysed using Ingenuity VA version 6.0.20200522 (Qiagen, www.qiagen.com/ingenuity). Variants were filtered first for high confidence using all available options (default settings). We also required

variants to have a QD of 2 or more and a MQ of at least 40. Variants with a population allele frequency of 0.5% or more were removed using the global 1000 Genome Frequency (phase3v5b) and gnomAD (2.1.1). For the latter database, the "subpopulation with the highest allele frequency" setting was employed. We then filtered for variants that were frameshifts, in-frame indels, stop-codon changes, missense, canonical splice sites (+/- 2bp into intron) or predicted to disrupt splicing by MaxEntScan. Finally, we then filtered for variants that were called homozygous in the proband and present in the regions of autozygosity shared between the proband and the uncle.

Supplementary Case Reports

Available for all families as a separate docx file.

Supplementary Videos

Supplementary videos are available online (https://academic.oup.com/brain) for 8/17 affected individuals as mp4 files and all obtained with patient or parental consent.

Supplementary Video 1 shows the 50-year-old proband from Family 1. She has high arched feet, proximal lower limbs muscle weakness and is unable to stand up from squat. She cannot walk on her hills. There are asymmetric and poorly reactive pupils to direct light and no accommodation-convergence reflex. Mild fasciculations in the resting tongue. Mild distal>proximal weakness in the upper limbs with hand muscle wasting can be observed. There is also mild distal hand tremor with arms outstretched. Proximal more than distal lower limb weakness can be seen with mild wasting at the level of lower thigh muscles. There are Achilles tendon contractures with suppressed and absent tendon reflexes. Babinski sign is negative. Muscle tone from the lower limbs is normal and sensory examination is intact.

Supplementary Video 2 shows a 70-year-old man (Family 3) walking backward and forward without shoes. He has bilateral proximal muscle weakness affecting his gait. Some distal weakness in the lower limbs can also be noticed. He has forward stood posture and foot deformities clear seen in the left foot which is inverted.

Supplementary Video 3 shows a 70-years-old female (Family 8), who is able to stand up from the chair with arms crossed over chest. Upon standing and walking lumbar hyperlordosis and gait unsteadiness can be noticed. She is unable to perform tandem walking.

Supplementary Video 4 displays a 6-year-old girl (Family 10) running backward and forward with a wide gait. She slightly inverts her right foot when running.

Supplementary Video 5 shows an 8-year-old boy (Family 11) running backward and forward with a wide gait. His left foot is inverted on running.

Supplementary Video 6 shows wheelchair transfer for proband (elder of two MZ twins) from Family 12.

Supplementary Video 7 displays the 11-year-old proband from Family 13. He has bilateral hip and knee flexion contractures with forward stood posture and proximal myopathic gait. Muscle bulk is generally reduced which is more predominant in the distal and proximal lower limbs. He has scapular winging.

Supplementary Video 8 shows a 24-year-old female (Family 15) walking backward and forward. She has left prosthetic leg and walks with right foot drop.

Supplementary Discussion

We hypothesise that poor coverage may be a significant factor in explaining why the founder mutation in *VWA1* has not previously been reported. The first coding exon where the 10bp insertion lies is not typically captured very well by exome sequencing studies. Consequently, the variant is seen at 0.053% (15/28,196) in the genome sequences from gnomAD v2.1.1 but was not detected in the exome data from the same resource (0/16,854). Further interrogation of the coverage statistics available from for gnomAD v2.1.1 shows that <10% of exome datasets have 10x or more coverage at this locus. This is in contrast to the genomes available from the same resource, for which ~90% have 10x or more (Supplementary Fig. 13). Low efficiency of capture is likely due to the high GC content (>80%); this is a well-established problem associated with hybridisation-based capture methods (Clark *et al.*, 2011). Sequence depth for genome sequencing is less influenced by GC content and so is more uniform across *VWA1*. This study therefore acts as an exemplar highlighting the advantages of full genome sequencing.

Even when genome sequencing has generated sufficient coverage across the locus, quality metrics for the founder variant were variable. Although both small variant callers employed as part of the 100KGP project called the founder mutation, for 7/10 families Platypus either missed the variant entirely or else flagged one of the variants as being a low confidence call

("badReads") such that *VWA1* was filtered out in the subsequent tiering pipeline. In contrast, ISAAC called the insertion with a PASS flag in all families. The inserted sequence is already present twice in the reference sequence (Supplementary Fig. 13) which means the insertion could be also classed as a repeat expansion. Repeat expansions are known to be difficult to detect with short-read data. Although dedicated software is available (Bahlo *et al.*, 2018), these tools are typically optimised for larger expansions of repeats for which the repeat unit is 3-6bp in size.

Supplementary References

Bahlo M, Bennett MF, Degorski P, Tankard RM, Delatycki MB, Lockhart PJ. Recent advances in the detection of repeat expansions with short-read next-generation sequencing. F1000Res 2018; 7.

Chow CY, Zhang Y, Dowling JJ, Jin N, Adamska M, Shiga K, *et al.* Mutation of FIG4 causes neurodegeneration in the pale tremor mouse and patients with CMT4J. Nature 2007; 448(7149): 68-72.

Clark MJ, Chen R, Lam HY, Karczewski KJ, Chen R, Euskirchen G, *et al.* Performance comparison of exome DNA sequencing technologies. Nat Biotechnol 2011; 29(10): 908-14.

Cortese A, Zhu Y, Rebelo AP, Negri S, Courel S, Abreu L, *et al.* Biallelic mutations in SORD cause a common and potentially treatable hereditary neuropathy with implications for diabetes. Nat Genet 2020.

Cottenie E, Kochanski A, Jordanova A, Bansagi B, Zimon M, Horga A, *et al.* Truncating and missense mutations in IGHMBP2 cause Charcot-Marie Tooth disease type 2. Am J Hum Genet 2014; 95(5): 590-601.

Cuesta A, Pedrola L, Sevilla T, Garcia-Planells J, Chumillas MJ, Mayordomo F, *et al.* The gene encoding ganglioside-induced differentiation-associated protein 1 is mutated in axonal Charcot-Marie-Tooth type 4A disease. Nat Genet 2002; 30(1): 22-5.

Delague V, Jacquier A, Hamadouche T, Poitelon Y, Baudot C, Boccaccio I, *et al.* Mutations in FGD4 encoding the Rho GDP/GTP exchange factor FRABIN cause autosomal recessive Charcot-Marie-Tooth type 4H. Am J Hum Genet 2007; 81(1): 1-16.

Giannikou K, Fryssira H, Oikonomakis V, Syrmou A, Kosma K, Tzetis M, *et al.* Further delineation of novel 1p36 rearrangements by array-CGH analysis: narrowing the breakpoints and clarifying the "extended" phenotype. Gene 2012; 506(2): 360-8.

Grohmann K, Schuelke M, Diers A, Hoffmann K, Lucke B, Adams C, *et al.* Mutations in the gene encoding immunoglobulin mu-binding protein 2 cause spinal muscular atrophy with respiratory distress type 1. Nat Genet 2001; 29(1): 75-7.

Heilstedt HA, Ballif BC, Howard LA, Kashork CD, Shaffer LG. Population data suggest that deletions of 1p36 are a relatively common chromosome abnormality. Clin Genet 2003; 64(4): 310-6.

Martin AR, Williams E, Foulger RE, Leigh S, Daugherty LC, Niblock O, *et al.* PanelApp crowdsources expert knowledge to establish consensus diagnostic gene panels. Nat Genet 2019; 51(11): 1560-5.

Parsons DW, McAndrew PE, Monani UR, Mendell JR, Burghes AH, Prior TW. An 11 base pair duplication in exon 6 of the SMN gene produces a type I spinal muscular atrophy (SMA) phenotype:

further evidence for SMN as the primary SMA-determining gene. Hum Mol Genet 1996; 5(11): 1727-32.

Richards S, Aziz N, Bale S, Bick D, Das S, Gastier-Foster J, *et al.* Standards and guidelines for the interpretation of sequence variants: a joint consensus recommendation of the American College of Medical Genetics and Genomics and the Association for Molecular Pathology. Genet Med 2015; 17(5): 405-24.

Rimmer A, Phan H, Mathieson I, Iqbal Z, Twigg SRF, Consortium WGS, *et al.* Integrating mapping-, assembly- and haplotype-based approaches for calling variants in clinical sequencing applications. Nat Genet 2014; 46(8): 912-8.

Senderek J, Bergmann C, Stendel C, Kirfel J, Verpoorten N, De Jonghe P, *et al.* Mutations in a gene encoding a novel SH3/TPR domain protein cause autosomal recessive Charcot-Marie-Tooth type 4C neuropathy. Am J Hum Genet 2003; 73(5): 1106-19.

Vijzelaar R, Snetselaar R, Clausen M, Mason AG, Rinsma M, Zegers M, *et al.* The frequency of SMN gene variants lacking exon 7 and 8 is highly population dependent. PLoS One 2019; 14(7): e0220211.
